# Supplementary material for: Opposing Development of Cytotoxic and Follicular Helper CD4 T Cells Controlled by the TCF-1-Bcl6 Nexus
Source: Cell Rep. 2016 Nov 1;17(6):1571–83. doi: 10.1016/j.celrep.2016.10.013 (PMC5149578; doi:10.1016/j.celrep.2016.10.013)
Supplement: Document S1. Supplemental Experimental Procedures, Figures S1–S15, and Table S2 [file mmc1.pdf]

Cell Reports, Volume *17*

Supplemental Information

Opposing Development of Cytotoxic and Follicular Helper CD4 T Cells Controlled by the TCF-1-Bcl6 Nexus

Tiziano Donnarumma, George R. Young, Julia Merkenschlager, Urszula Eksmond, Nadine Bongard, Stephen L. Nutt, Claude Boyer, Ulf Dittmer, Vu Thuy Khanh Le-Trilling, Mirko Trilling, Wibke Bayer, and George Kassiotis

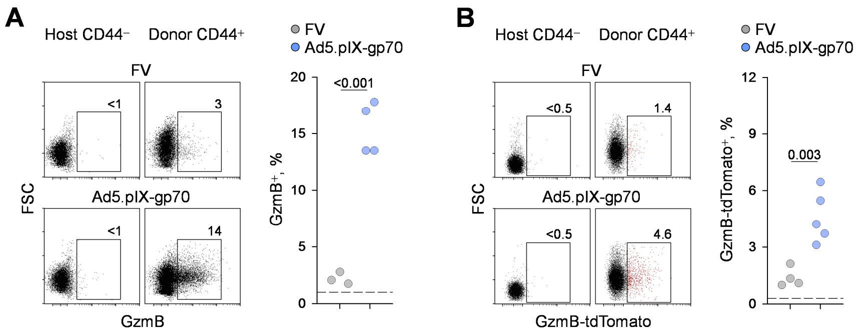

Figure S1. Related to Figure 1; Detection of GzmB production without *in vitro* restimulation

(A) Flow cytometric detection of intracellular GzmB in host naïve ($CD44^-$) or env-reactive ($CD44^+$) donor EF4.1 $CD4^+$ T cells (*left*) and frequency of $GzmB^+$ cells in env-reactive donor EF4.1 $CD4^+$ T cells (*right*) in the spleens of recipient mice, 7 days after adoptive transfer and FV infection or Ad5.pIX-gp70 immunization, without an *in vitro* restimulation step. In the scatter plot, each symbol is an individual recipient. One representative of two experiments is shown.

(B) Flow cytometric detection of tdTomato fluorescence from a chimeric GzmB protein in host naïve ($CD44^-$) or env-reactive ($CD44^+$) donor *Gzmb*^{tdTom} EF4.1 $CD4^+$ T cells (*left*) and frequency of GzmB-tdTomato$^+$ cells in env-reactive donor *Gzmb*^{tdTom} EF4.1 $CD4^+$ T cells (*right*) in the spleens of recipient mice, 7 days after adoptive transfer and FV infection or Ad5.pIX-gp70 immunization. In the scatter plot, each symbol is an individual recipient from a single experiment.

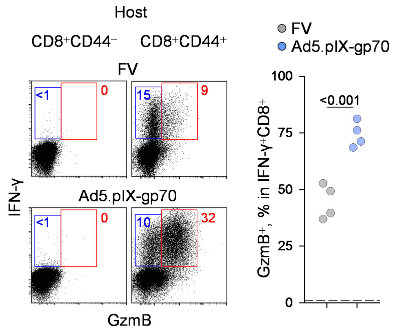

Figure S2. Related to Figure 1; CD8$^+$ CTL development depends on infecting virus

(A) Flow cytometric detection of intracellular GzmB and IFN-γ in naïve (CD44$^-$) or memory-phenotype (CD44$^+$) host CD8$^+$ T cells (*left*) and frequency of GzmB$^+$ cells in CD44$^+$IFN-γ$^+$CD8$^+$ T cells (*right*) in the spleens of mice, 7 days after FV infection or Ad5.pIX-gp70 immunization. In the scatter plot, each symbol is an individual recipient. One representative of three experiments is shown.

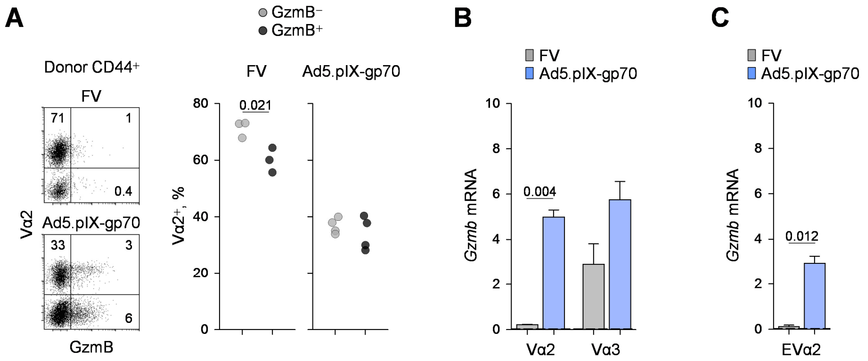

Figure S3. Related to Figure 1; Effect of TCR avidity on $CD4^+$ CTL differentiation

(A) Flow cytometric detection of intracellular GzmB according to TCR Vα2 expression in env-reactive ($CD44^+$) donor EF4.1 $CD4^+$ T cells (*left*) and frequency of $Vα2^+$ cells in the $GzmB^-$ and $GzmB^+$ subsets of env-reactive donor EF4.1 $CD4^+$ T cells in the spleens of recipient mice, 7 days after adoptive transfer and FV infection or Ad5.pIX-gp70 immunization. In the scatter plot, each symbol is an individual recipient.

(B) Expression of *Gzmb*, relative to *Hprt*, assessed by qRT-PCR in the Vα2 and Vα3 subsets of env-reactive donor EF4.1 $CD4^+$ T cells purified from the spleens of recipient mice, 7 days after adoptive transfer and FV infection or Ad5.pIX-gp70 immunization. Plotted are the mean values (±SEM) of 2 technical replicates, from 2 experiments with 4 mice per group per experiment.

(C) Expression of *Gzmb*, relative to *Hprt*, assessed by qRT-PCR in env-reactive donor monoclonal EVα2 $CD4^+$ T cells purified from the spleens of recipient mice, 7 days after adoptive transfer and FV infection or Ad5.pIX-gp70 immunization. Plotted are the mean values (±SEM) of 2 technical replicates, from 1 experiment with 5 mice per group.

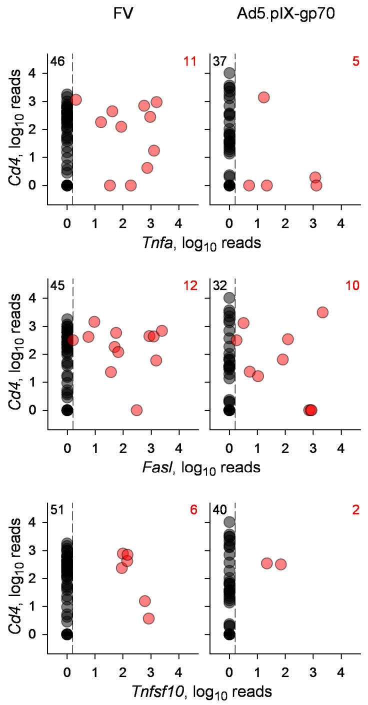

Figure S4. Related to Figure 2; Comparable *Tnfa*, *Fasl* and *Tnfsf10* induction in CD4^{+} CTLs by FV infection and Ad5.pIX-gp70 immunization

Expression of *Tnfa, Fasl and Tnfsf10*, assessed by single-cell RNA sequencing, in env-reactive donor EF4.1 CD4^{+} T cells purified from the spleens of recipient mice, 7 days after adoptive transfer and FV infection or Ad5.pIX-gp70 immunization.

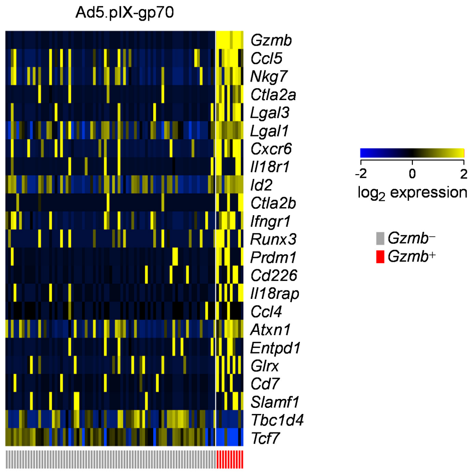

Figure S5. Related to Figure 2; Transcriptional signature of CD4$^+$ CTLs induced by Ad5.pIX-gp70 immunization

Heat-map of gene expression, assessed by single-cell RNA sequencing, comparing *Gzmb*$^+$ and *Gzmb*$^-$ subsets in env-reactive donor EF4.1 CD4$^+$ T cells purified from the spleens of recipient mice, 7 days after adoptive transfer and priming. CD4$^+$ T cells only from Ad5.pIX-gp70 immunization are included. Numbers of *Gzmb*$^+$ cells induced by FV infection were too low to allow for a similar comparison.

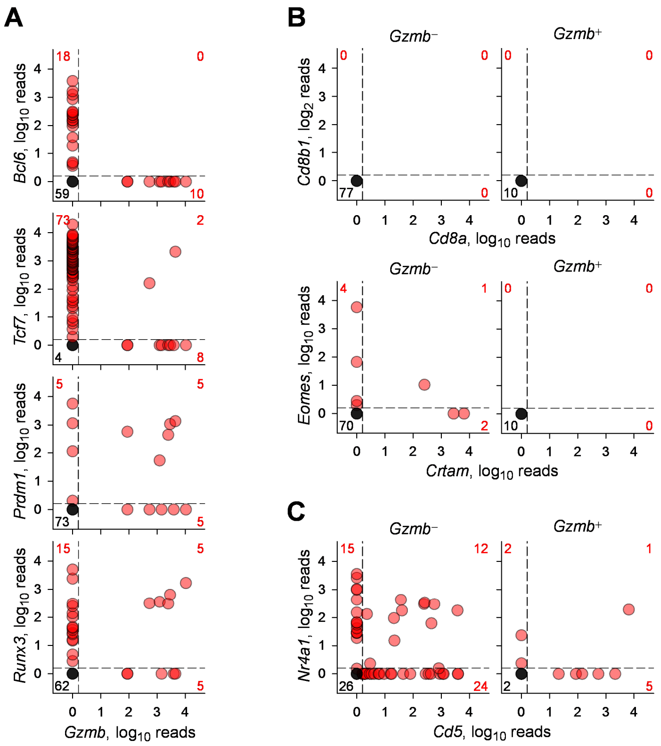

Figure S6. Related to Figure 2; Transcriptional comparison of single *Gzmb*⁺ and *Gzmb*⁻ CD4⁺ T cells

(A) Expression of *Bcl6*, *Tcf7*, *Prdm1* and *Runx3*, according to *Gzmb* expression, assessed by single-cell RNA sequencing, in env-reactive donor EF4.1 CD4⁺ T cells purified from the spleens of recipient mice, 7 days after adoptive transfer and Ad5.pIX-gp70 immunization.

(B) Expression of *Cd8a* and *Cd8b1* (*top*) and *Crtam and Eomes* (*bottom*), assessed by single-cell RNA sequencing, separately in *Gzmb*⁺ and *Gzmb*⁻ env-reactive donor EF4.1 CD4⁺ T cells purified from the spleens of recipient mice, 7 days after adoptive transfer and Ad5.pIX-gp70 immunization.

(C) Expression of *Cd5* and *Nr4a1* in the same cells as in B.

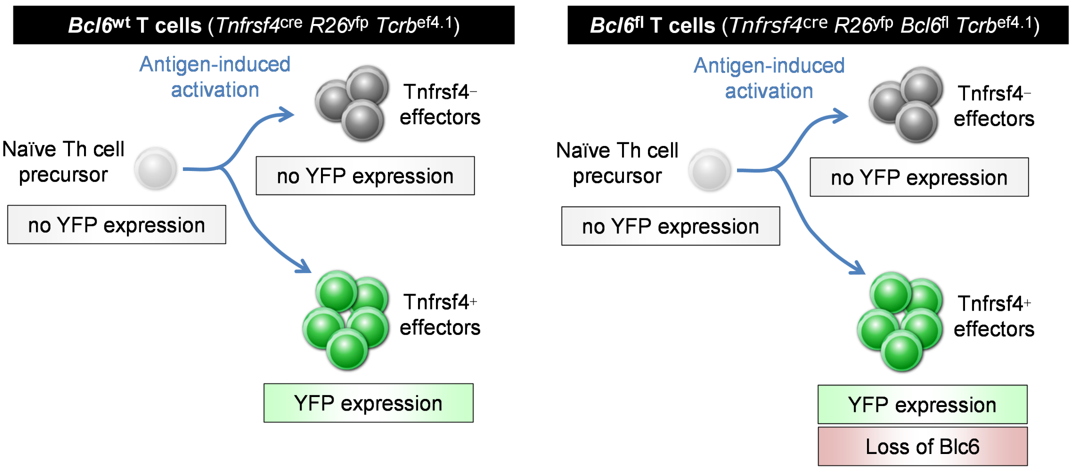

Figure S7. Related to Figure 3; Strategy for conditional deletion of *Bcl6* in effector CD4^{+} T cells

Conditional ablation of *Bcl6* in env-specific effector CD4^{+} T cells is achieved by expression of Cre in donor CD4^{+} T cells, transferred into wild-type recipients, under the control of the *Tnfrsf4* promoter (*Tnfrsf4*^{Cre}). Cells that activated Cre are identified by the use of a conditional YFP reporter (*Gt(ROSA)26Sor*^{YFP}). Naïve Th cell precursors, which do not express *Tnfrsf4*, are unaffected and they neither gain YFP nor lose Bcl6.

In control *Bcl6*^{wt} mice (*left*), antigen-induced activation of env-specific naïve Th cell precursors leads to *Tnfrsf4* transcription in a proportion (70-80%) of cells. In turn, this drives *Cre* transcription, ultimately leading to YFP expression. In these mice, YFP marks the cells that activate the *Tnfrsf4* promoter.

In *Bcl6*^{fl} mice (*right*), additionally carrying the Cre-conditional *Bcl6*^{fl} allele, antigen-induced activation of env-specific naïve Th cell precursors leads not only to YFP expression, but also to loss of Bcl6 in the same cells.

The YFP-expressing populations between the two types of donors represent CD4^{+} T cells at the same state of activation (but differing in Bcl6 expression) and are directly comparable.

Both types of donor CD4^{+} T cells also contain YFP-negative effector cells that do not activate the *Tnfrsf4* promoter and therefore do not lose the capacity to express Bcl6. These YFP-negative populations additionally serve as internal controls.

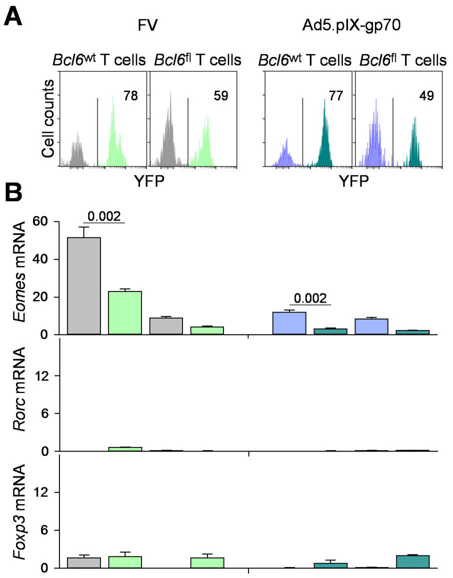

Figure S8. Related to Figure 3; Bcl6 suppresses CD4⁺ CTL development

(A) Delineation of env-reactive donor EF4.1 CD4$^+$ T cells according to YFP expression as in Fig. S7.

(B) Expression of the indicated gene, relative to *Hprt*, assessed by qRT-PCR in the respective bulk subset of env-reactive donor EF4.1 CD4$^+$ T cells shown immediately above in A. Plotted are the mean values (±SEM) of 2 technical replicates, from 2 experiments with 5 mice per group per experiment.

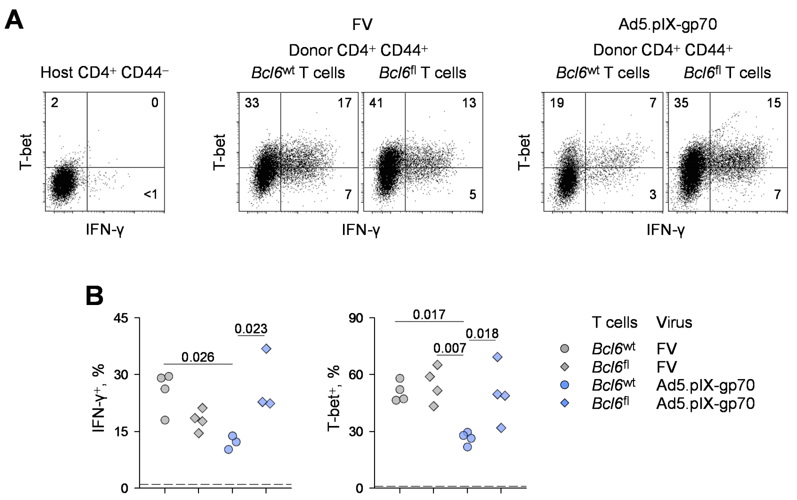

Figure S9. Related to Figure 3; Effect of *Bcl6* deletion on CD4$^+$ effector development

(A) Flow cytometric detection of intracellular IFN-γ and T-bet expression in host naïve (CD44$^-$) or env-reactive (CD44$^+$) *Bcl6*^{wt} or *Bcl6*^{fl} donor EF4.1 CD4$^+$ T cells, in the spleens of recipient mice, 7 days after adoptive transfer and FV infection or Ad5.pIX-gp70 immunization.

(B) Frequency of IFN-γ$^+$ and T-bet$^+$ cells in env-reactive *Bcl6*^{wt} or *Bcl6*^{fl} donor EF4.1 CD4$^+$ T cells from the same recipients. Each symbol is an individual mouse from one representative of two experiments.

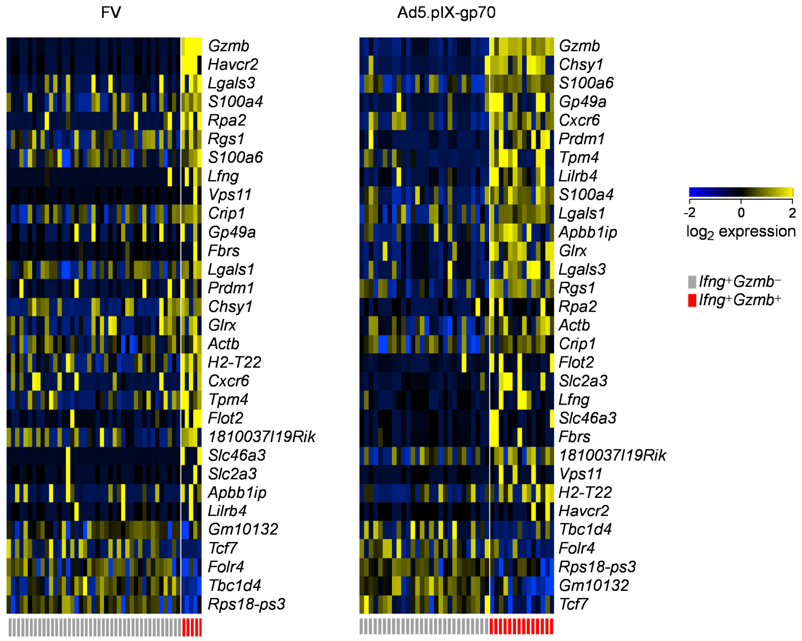

Figure S10. Related to Figure 5; CD4$^+$ CTL and Th1 cells are transcriptionally distinct: effect of priming virus

Heat-maps of gene expression, assessed by single-cell RNA sequencing, comparing *Ifng*$^+$*Gzmb*$^+$ and *Ifng*$^+$*Gzmb*$^-$ subsets of env-reactive donor EF4.1 CD4$^+$ T cells purified from the spleens of recipient mice, 7 days after adoptive transfer either FV infection (p=0.064) (*left*) or Ad5.pIX-gp70 immunization (p=0.00154) (*right*). Both *Bcl6*^{wt} and *Bcl6*^{fl} CD4$^+$ T cells are included.

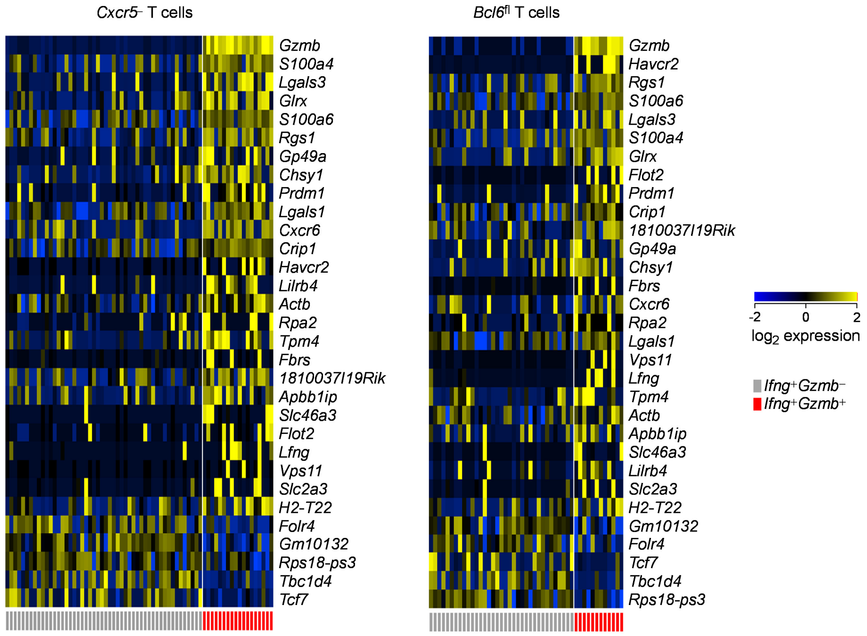

Figure S11. Related to Figure 5; CD4$^+$ CTL and Th1 cells are transcriptionally distinct: exclusion of Tfh cells

Heat-maps of gene expression, assessed by single-cell RNA sequencing, comparing *Ifng*$^+$*Gzmb*$^+$ and *Ifng*$^+$*Gzmb*$^-$ subsets of env-reactive donor EF4.1 CD4$^+$ T cells purified from the spleens of recipient mice, 7 days after adoptive transfer and priming. Cells from both FV infection and Ad5.pIX-gp70 immunization are included. Tfh cells were omitted from this analysis either by excluding cells that express *Cxcr5* (p=0.0038) (*left*) or by including only cells that have conditionally lost *Bcl6* (p=0.0345) (*right*), and therefore the ability to differentiate into Tfh cells.

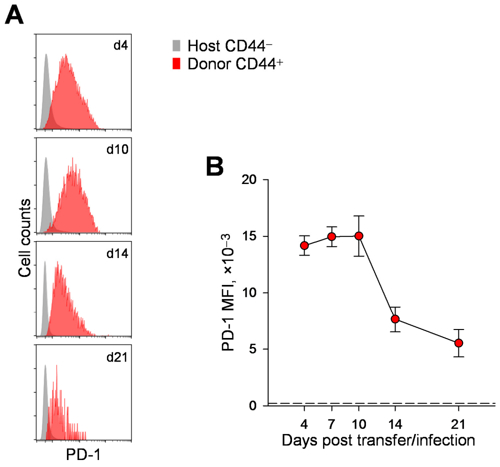

Figure S12. Related to Figure 6; Time course of PD-1 expression in CD4$^+$ T cells responding to FV infection

(A) Flow cytometric detection of surface PD-1 expression in host naïve (CD44$^-$) or env-reactive (CD44$^+$) donor EF4.1 CD4$^+$ T cells, in the spleens of recipient mice, at the indicated days after adoptive transfer and FV infection.

(B) MFI of PD-1 staining in the same cells as in A. Plotted are the mean values (±SEM) from 1 experiment with 4 mice per time-point.

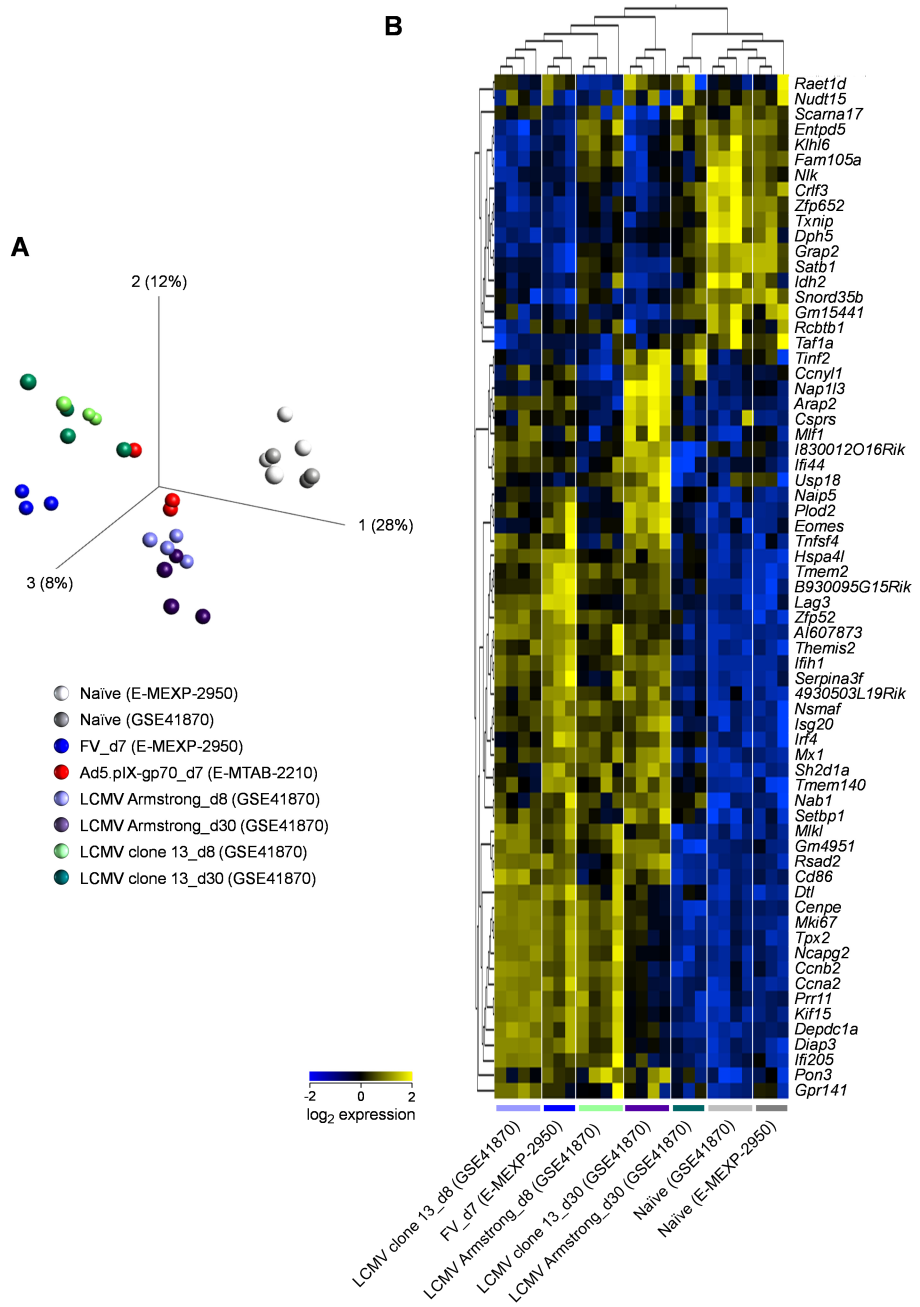

Figure S13. Related to Figure 6; Comparison of FV-primed effector and exhausted CD4^{+} T cells

(A) Principal component analysis (PCA) of the transcriptional profiles of naïve and FV-primed effector (d7) EF4.1 CD4^{+} T cells (study E-MEXP-2950), Ad5.pIX-gp70-primed effector (d7) EF4.1 CD4^{+} T cells (study E-MTAB-2210) and naïve, effector (d8) and exhausted (d30) CD4^{+} T cells primed by either LCMV Armstrong or clone 13 (study GSE41870). LCMV clone 13-primed CD4^{+} T cells on day 30 of infection typify the exhaustion profile. All transcriptional profiles were obtained with the Affymetrix Mouse Gene 1.0 ST Array and can be accessed with the indicated accession numbers.

(B) Hierarchical clustering of the same samples according to the expression of the list of genes reported to signify the exhaustion phenotype (Crawford 2014; Table S3). FV-primed effectors cluster together with other effector subsets.

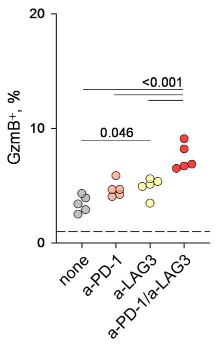

Figure S14. Related to Figure 6; PD-1 and LAG3 cooperatively inhibit CD4$^+$ CTL development

Frequency of intracellular GzmB$^+$ cells in bulk env-reactive donor EF4.1 CD4$^+$ T cells in the spleens of recipient mice, 7 days after adoptive transfer and FV infection. The indicated groups additionally received treatment with either PD-1 or LAG3 blocking antibodies separately or combined. Each symbol is an individual recipient.

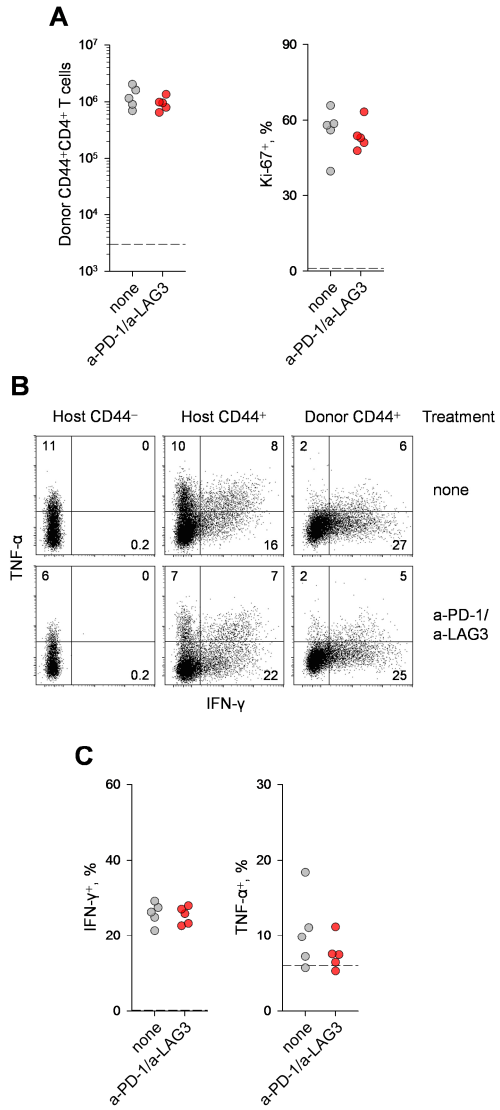

Figure S15. Related to Figure 6; Effect of PD-1 and LAG3 blockade on CD4$^+$ effector development

(A) Absolute numbers of env-reactive donor EF4.1 CD4$^+$ T cells (*left*) and frequency of Ki67$^+$ cells within these cells (*right*) recovered from the spleens of recipient mice, 7 days after adoptive transfer and FV infection, in the presence or absence of PD-1 or LAG3 blocking antibodies.

(B) Flow cytometric detection of intracellular IFN-γ and TNF-α expression in host naïve (CD44$^-$) or env-reactive (CD44$^+$) donor EF4.1 CD4$^+$ T cells, in the spleens of recipient mice, 7 days after adoptive transfer and FV infection, in the presence or absence of PD-1 or LAG3 blocking antibodies.

(C) Frequency of IFN-γ$^+$ and TNF-α$^+$ cells in the same cells as in B. In A and C, each symbol is an individual mouse and dashed lines represents host naïve CD4$^+$ T cells.

Supplementary Table 1: Differences in gene expression between Gzmb+ and Gzmb- cells.

Please see Excel file

Supplementary Table 2: PCR primers used in this study

Gene	Forward primer	Reverse primer
Zbtb7b	ATGGGATTCCAATCAGGTCA	TTCTTCCTACACCCTGTGCC
Runx3	ACAGCATCTTTGACTCCTTCC	TGTTCTCGCCCATCTTGC
Tcf7	CAATCTGCTCATGCCCTACC	CTTGCTTCTGGCTGATGTCC
Gzmb	CCTCCTGCTACTGCTGACCT	TAAGGCCATGTAGGGTCGAG
Prdm1	ACATAGTGAACGACCACCCCTG	CTTACCACGCCAATAACCTCTTTG
Bcl6	CCTGTGAAATCTGTGGCACTCG	CGCAGTTGGCTTTTGTGAC
Gata3	ACAGAAGGCAGGGAGTGTGTGAAC	TTTTATGGTAGAGTCCGCAGGC
Tbx21	CAATGTGACCCAGATGATCG	GCGTTCTGGTAGGCAGTCAC
Ifng	GATGCATTCATGAGTATTGCCAAGT	GTGGACCACTCGGATGAGCTC
Hprt	TTGTATACCTAATCATTATGCCGAG	CATCTCGAGCAAGTCTTTCA

SUPPLEMENTAL EXPERIMENTAL PROCEDURES

Retroviral infection and immunization

The FV used in this study was a retroviral complex of a replication-competent B-tropic F-MLV and a replication-defective spleen-focus forming virus (SFFV). Stocks were propagated *in vivo* and prepared as 10% w/v homogenate from the spleen of 12-day infected BALB/c mice, as previously described (Marques et al., 2008). Mice received an inoculum of ~1,000 spleen focus-forming units of FV by intravenous injection. Stocks of F-MLV-N helper virus were grown in *Mus dunni* fibroblast cells. Mice received an inoculum of ~10^4 infectious units of F-MLV-N by intravenous injection. Ad5.pIX-gp70 stocks were prepared at a titer of 9×10^9 viral genomes ml^{-1} by infection of 293A cells (Thorborn et al., 2014). Approximately 5×10^8 Ad5.pIX-gp70 viral genomes per mouse were administered intravenously. The mCMV vector expressing F-MLV env (mCMV.env) was constructed by inserting the F-MLV *env* open reading frame, under the control of the human CMV major IE promoter/enhancer, into the m157 open reading frame of mCMV in the MCK-2 repaired background cloned into a bacterial artificial chromosome (BAC) (Jordan et al., 2011). The recombinant mCMV.env was reconstituted from BAC DNA by transfection into permissive fibroblasts. Persistence of the mCMV vector, as well as retention of the F-MLV *env* transgene, were confirmed by PCR in mCMV.env genomes isolated from the salivary glands of infected mice at 21 days post infection. Mice received 2×10^5 plaque-forming units of mCMV.env intraperitoneally. All stocks were free of Sendai virus, Murine hepatitis virus, Parvoviruses 1 and 2, Reovirus 3, Theiler's murine encephalomyelitis virus, Murine rotavirus, Ectromelia virus, Murine cytomegalovirus, K virus, Polyomavirus, Hantaan virus, Murine norovirus, Lymphocytic

choriomeningitis virus, Murine adenoviruses FL and K87, *Mycoplasma* sp. and Lactate dehydrogenase elevating virus. FBL-3 tumor challenge was carried out by intravenous injection of 3×10^6 FBL-3 cells (Klarnet et al., 1989). For peptide immunization, mice received an intraperitoneal injection of a total of 12.5 nmol of synthetic $env_{122-141}$ peptide mixed in Sigma Adjuvant System. Where indicated, recipient mice also received blocking antibodies against PD-1 (10 mg/kg, clone RMP1) and LAG3 (20mg/kg, clone C9B7W) (both from BioXCell, West Lebanon, NH, USA) injected intraperitoneally on days 0, 2 and 4 of FV infection.

Single-cell RNA sequencing

Cells were then applied to the Fluidigm C1 chip (Fluidigm, San Francisco, CA, USA) and captures were imaged for manual grading. Captures were split into 5 categories: no capture (0), debris (D), single cell (1), multiple cells (M), and non-standard single cell (NS - including large, small, potentially damaged or dying). Libraries were constructed using the SMART-Seq v4 Ultra Low RNA Kit for the Fluidigm C1 System (Clontech, Mountain View, CA, USA), following the manufacturer's instructions, and validated with the use of spiked-in RNA species (Ambion ArrayControl RNA Spikes, Thermo Fisher Scientific, Waltham, MA USA). Reads (2×50 bp paired-end), generated with the Illumina HiSeq 2000 (Illumina, San Diego, CA, USA), were adapter- and quality-trimmed using Trimmomatic 0.32 (Bolger et al., 2014). Resulting reads pairs with a read <30 nucleotides in length were discarded, resulting in a median pair retention of 94%. Pairs were mapped to the mouse genome (GRCm38.78) with the splice-aware aligner HISAT (Kim et al., 2015) and gene-level counts were produced with featureCounts (Liao et al., 2014). To streamline downstream analysis, any genes not found

expressed in at least 1% of samples were discarded, resulting in 11463 genes in the subsequent analysis. Whilst only morphologically normal single cells are included in the '1' capture category, any library preparations may suffer as a result of poor lysis of the cell, or if, whilst morphologically normal, a cell was dead or dying. To prevent such libraries from impacting the analysis and to allow inclusion of transcriptionally similar cells from the 'NS' capture category, a classifier was build using all cells. DESeq2 (Love et al., 2014) was used for initial library size normalization and individual differential expression (DE) analysis of '0' and 'D' captures against combined '1', 'NS', and 'M' captures. The intersect of the top 250 DE genes for these capture categories was taken, giving a list of 76 genes that were used to build a supervised SVM classifier using the R e1071 package (wrapping LIBSVM) (Chang and Lin, 2011) that was auto-tuned to the data. Excluding '0', 'D', and 'M' capture categories, the SVM was applied to '1' and 'NS' cells, providing a numerical likelihood of a sample having better correspondence to a '0' or 'D' capture. Any cells with correspondence 50% or larger were excluded from further analysis. Plots of percentage alignment against number of genes identified for cells passing and failing these filters confirmed that excluded cells frequently had poor alignment rates and lower numbers of genes identified as expressed, although overall read count did not correspond to either of these metrics. Subsequently, samples with <15% alignment rate and those with <1000 genes identified as expressed were additionally excluded. Retained cells from the '1' and 'NS' capture categories were again passed to DESeq2 and DE analysis was performed between various conditions. Normalised count data was exported at this point for further analysis and plotting using Qlucore Omics Explorer (Qlucore, Lund, Sweden). The European Nucleotide Archive (ENA) accession number for the single-cell RNA sequences reported in this paper is PRJEB14043.

Cytotoxicity assays

In vivo cytotoxicity was assessed by the specific killing of peptide-pulsed targets of $CD4^+$ CTLs. Single-cell suspensions were prepared from the spleens and lymph nodes of donor $CD45.1^+$ or $CD45.2^+$ WT mice and B cells ($B220^+$ cells) were purified using immunomagnetic positive selection (StemCell Technologies). B cells from only one type of donor were incubated *in vitro* for 2 hours with 1µM $env_{122\text{-}141}$ peptide. After washing, pulsed and non-pulsed B cells from the two types of donor were mixed at equal ratios and co-injected into the indicated recipients (5×10^6 of each per recipient). The ratio between pulsed and non-pulsed B cells was measured by flow cytometry in the spleens of recipients 24 hours after B cell transfer. The percentage difference in this ratio was expressed as percentage killing. *In vitro* cytotoxicity was measured using the GranToxiLux-PLUS kit (Oncoimmunin, Gaithersburg, MD, USA), according to the manufacturer's instructions. Briefly, WT splenocytes were used as target cells and were either incubated *in vitro* for 2 hours with 1µM $env_{122\text{-}141}$ peptide (pulsed) or not (non-pulsed). Effector EF4.1 $CD4^+$ T cells were purified by cell sorting from the spleens of FV infected or Ad5.pIX-gp70 vaccinated recipients on day 7 of the response. Target cells were identified by labelling with a target fluorescent probe (TFL-4) and with a nuclear fluorescent labelling probe (NFL1), to exclude cells that had died before the start of the assay. Effector and target cells were mixed at a ratio of 5:1 and co-incubated in the presence of a FITC-conjugated GzmB substrate for 2 hours. Cytotoxic activity was detected by the cleavage of the substrate, which released FITC and thus rendered target cells fluorescent.

SUPPLEMENTAL REFERENCES

Bolger, A.M., Lohse, M., and Usadel, B. (2014). Trimmomatic: a flexible trimmer for Illumina sequence data. Bioinformatics *30*, 2114-2120.

Chang, C.-C., and Lin, C.-J. (2011). LIBSVM: A library for support vector machines. ACM Trans. Intell. Syst. Technol. *2*, 1-27.

Jordan, S., Krause, J., Prager, A., Mitrovic, M., Jonjic, S., Koszinowski, U.H., and Adler, B. (2011). Virus progeny of murine cytomegalovirus bacterial artificial chromosome pSM3fr show reduced growth in salivary Glands due to a fixed mutation of MCK-2. J. Virol. *85*, 10346-10353.

Kim, D., Langmead, B., and Salzberg, S.L. (2015). HISAT: a fast spliced aligner with low memory requirements. Nat. Methods *12*, 357-360.

Klarnet, J.P., Kern, D.E., Okuno, K., Holt, C., Lilly, F., and Greenberg, P.D. (1989). FBL-reactive CD8+ cytotoxic and CD4+ helper T lymphocytes recognize distinct Friend murine leukemia virus-encoded antigens. J. Exp. Med. *169*, 457-467.

Liao, Y., Smyth, G.K., and Shi, W. (2014). featureCounts: an efficient general purpose program for assigning sequence reads to genomic features. Bioinformatics *30*, 923-930.

Love, M.I., Huber, W., and Anders, S. (2014). Moderated estimation of fold change and dispersion for RNA-seq data with DESeq2. Genome Biol. *15*, 550.

Marques, R., Antunes, I., Eksmond, U., Stoye, J., Hasenkrug, K., and Kassiotis, G. (2008). B lymphocyte activation by coinfection prevents immune control of friend virus infection. J. Immunol. *181*, 3432-3440.

Thorborn, G., Ploquin, M.J., Eksmond, U., Pike, R., Bayer, W., Dittmer, U., Hasenkrug, K.J., Pepper, M., and Kassiotis, G. (2014). Clonotypic composition of the CD4+ T cell response to a vectored retroviral antigen is determined by its speed. J. Immunol. *193*, 1567-1577.
