# Supplementary material for: Opposing Development of Cytotoxic and Follicular Helper CD4 T Cells Controlled by the TCF-1-Bcl6 Nexus
Source: Cell Rep. 2016 Nov 1;17(6):1571–83. doi: 10.1016/j.celrep.2016.10.013 (PMC5149578; doi:10.1016/j.celrep.2016.10.013)
Supplement: Document S2. Article plus Supplemental Information [file mmc3.pdf]

Cell Reports

Article

Opposing Development of Cytotoxic and Follicular Helper CD4 T Cells Controlled by the TCF-1-Bcl6 Nexus

Graphical Abstract

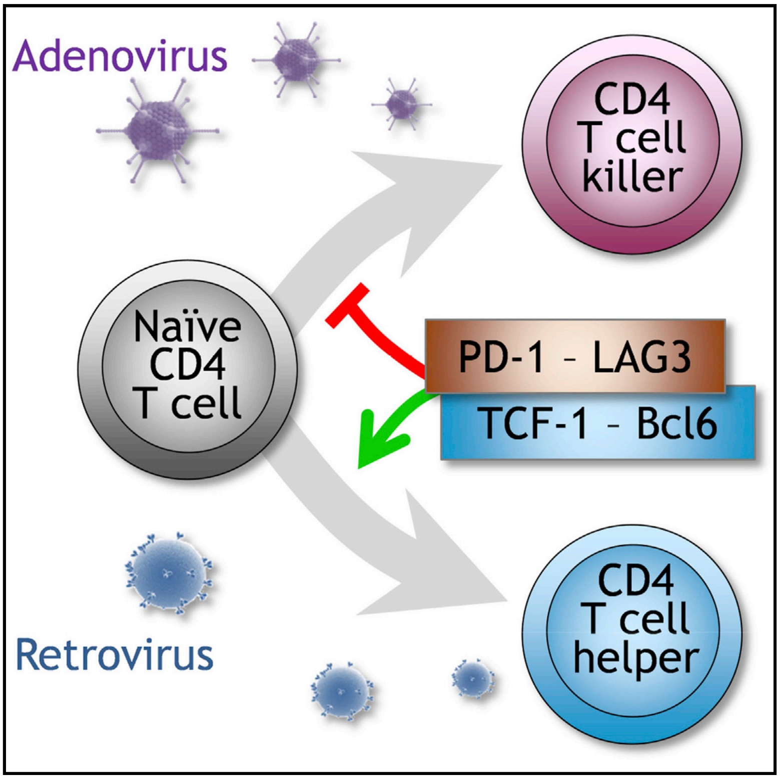

Highlights

- Adenoviruses prime CD4 T cells with CTL potential, but retroviruses do not
- CD4 CTLs are transcriptionally distinguishable from other Th cells
- The CD4 CTL program is the direct opposite of the Tfh program
- CD4 CTLs are restrained by the TCF-1-Bcl6 nexus and by PD-1 and LAG3

Authors

Tiziano Donnarumma, George R. Young, Julia Merkenschlager, ..., Mirko Trilling, Wibke Bayer, George Kassiotis

Correspondence

george.kassiotis@crick.ac.uk

In Brief

"Helper" CD4 T cells can also exhibit granzyme-mediated cytotoxicity. Donnarumma et al. investigate the conditions that induce CD4 CTLs and describe the dominant effect of the class of infecting virus. They uncover a unique transcriptional signature of CD4 CTLs and its multi-layered control.

Donnarumma et al., 2016, Cell Reports *17*, 1571–1583
November 1, 2016 © 2016 The Author(s).
http://dx.doi.org/10.1016/j.celrep.2016.10.013

OPEN
ACCESS
CellPress

Opposing Development of Cytotoxic and Follicular Helper CD4 T Cells Controlled by the TCF-1-Bcl6 Nexus

Tiziano Donnarumma,[1] George R. Young,[1,2] Julia Merkenschlager,[1] Urszula Eksmond,[1] Nadine Bongard,[4] Stephen L. Nutt,[5] Claude Boyer,[6] Ulf Dittmer,[4] Vu Thuy Khanh Le-Trilling,[4] Mirko Trilling,[4] Wibke Bayer,[4] and George Kassiotis[1,3,7,*]
[1]Retroviral Immunology
[2]Retrovirus-Host Interactions
The Francis Crick Institute, 1 Midland Road, London NW1 1AT, UK
[3]Department of Medicine, Faculty of Medicine, Imperial College London, London W2 1PG, UK
[4]Institute for Virology, University Hospital Essen, University Duisburg-Essen, 45122 Essen, Germany
[5]The Walter and Eliza Hall Institute of Medical Research, Parkville, VIC 3052, Australia
[6]Centre d'Immunologie de Marseille-Luminy (CIML), Aix-Marseille University, UM2, Marseille 13288, France
[7]Lead Contact
*Correspondence: george.kassiotis@crick.ac.uk
http://dx.doi.org/10.1016/j.celrep.2016.10.013

SUMMARY

CD4$^+$ T cells develop distinct and often contrasting helper, regulatory, or cytotoxic activities. Typically a property of CD8$^+$ T cells, granzyme-mediated cytotoxic T cell (CTL) potential is also exerted by CD4$^+$ T cells. However, the conditions that induce CD4$^+$ CTLs are not entirely understood. Using single-cell transcriptional profiling, we uncover a unique signature of Granzyme B (GzmB)$^+$ CD4$^+$ CTLs, which distinguishes them from other CD4$^+$ T helper (Th) cells, including Th1 cells, and strongly contrasts with the follicular helper T (Tfh) cell signature. The balance between CD4$^+$ CTL and Tfh differentiation heavily depends on the class of infecting virus and is jointly regulated by the Tfh-related transcription factors *Bcl6* and *Tcf7* (encoding TCF-1) and by the expression of the inhibitory receptors PD-1 and LAG3. This unique profile of CD4$^+$ CTLs offers targets for their study, and its antagonism by the Tfh program separates CD4$^+$ T cells with either helper or killer functions.

INTRODUCTION

CD4$^+$ TCRαβ T cells centrally orchestrate multiple arms of innate and adaptive immunity using distinct and, often, opposing effector and regulatory functions through the differentiation of distinguishable functional CD4$^+$ T cell subsets (O'Shea and Paul, 2010; Swain et al., 2012; Zhu et al., 2010). Several such functional subsets are now recognized, including the prototypic Th1 and Th2 subsets but also the Th17, follicular helper (Tfh), and regulatory T (Treg) subsets, each characterized by a well-defined transcriptional program (Crotty, 2014; O'Shea and Paul, 2010; Swain et al., 2012; Vinuesa et al., 2016; Zhu et al., 2010). Based on the increasingly appreciated diversity of CD4$^+$ T cell activities, additional functional subsets have been proposed. These include CD4$^+$ with cytotoxic T cell (CTL) potential, able to kill target cells through the release of granzyme-containing granules (Brown et al., 2016; Cheroutre and Husain, 2013; Swain et al., 2012).

Cytotoxicity is typically associated with CD8$^+$ T cells and natural killer (NK) cells and has not been conventionally considered a CD4$^+$ T cell function (Cullen et al., 2010). Indeed, "helper" and "cytotoxic" terms are often used to describe the major histocompatibility complex (MHC) class-II-restricted CD4$^+$ and MHC class-I-restricted CD8$^+$ TCRαβ T cell lineages, respectively. Commitment of developing thymocytes to the CD4$^+$ or CD8$^+$ lineage and acquisition of either helper or cytotoxic activity is controlled by the antagonistic transcription factors ThPOK and Runx3. ThPOK suppresses the cytotoxic program in CD4$^+$ thymocytes and mature T cells, whereas Runx3 promotes this program in CD8$^+$ T cells (Cheroutre and Husain, 2013).

Despite transcriptional repression of the cytotoxic program during CD4$^+$ T cell development, MHC class-II-restricted, cell-contact-dependent cytotoxicity has long been observed in a variety of conditions, both in humans and experimental animals (Brown, 2010; Brown et al., 2016; Cheroutre and Husain, 2013; Soghoian and Streeck, 2010; Swain et al., 2012; van de Berg et al., 2008). Although CD4$^+$ T cells can kill target cells also through surface expression of tumor necrosis factor (TNF) family members, including FasL and TRAIL, accumulated evidence has established their ability to develop and use granzyme-mediated cytotoxic activity (Brown et al., 2016; Cheroutre and Husain, 2013). Pivotal recent studies with a ThPOK-reporter mouse strain uncovered considerable plasticity of the "helper" program in CD4$^+$ T cells, with loss of ThPOK expression and transcriptional switch to the "cytotoxic" program. Indeed, a sizable fraction of CD4$^+$ T cells residing in the intestine as intraepithelial lymphocytes (IELs) downregulate the expression of *Zbtb7b*

This is an open access article under the CC BY license (http://creativecommons.org/licenses/by/4.0/).

(the gene encoding ThPOK) and acquire the expression of *Runx3* (Mucida et al., 2013; Reis et al., 2013). This transcriptional reprogramming is accompanied by the expression of genes more characteristic of the CD8$^+$ lineage, such as *Cd8a*, *Crtam*, and *Eomes*, and, importantly, also by the development of GzmB-mediated cytotoxic potential (Mucida et al., 2013; Reis et al., 2013).

These studies emphasize the similarities between CD8$^+$ T cells and reprogrammed CD4$^+$ CTLs and suggest that the latter subset should be viewed as distinct from other CD4$^+$ Th cell subsets (Cheroutre and Husain, 2013). Despite these significant advances, the factors that dictate CD4$^+$ CTL differentiation are still incompletely understood. Also unclear are the precise place of CD4$^+$ CTLs in the transcriptional spectrum of all Th cell subsets and whether the CD4$^+$ CTL program is compatible with other Th cell differentiation pathways.

Here, we investigated the priming requirements for CD4$^+$ CTLs and the signals that either promote or inhibit CD4$^+$ CTL differentiation. To overcome the lack of a reliable marker for the unambiguous identification of CD4$^+$ CTLs, we applied global transcriptional analysis of single CD4$^+$ T cells. We report that CD4$^+$ CTL differentiation strictly depends on the infecting or immunizing virus, with retroviral infection and adenovirus-based vaccination at the low and high ends of the spectrum, respectively. Moreover, our results uncovered regulation of the CD4$^+$ CTL program by expression of inhibitory receptors and direct antagonism by the Tfh program.

RESULTS

CD4$^+$ CTL Development Depends on Infecting Virus

We have previously described an adoptive transfer system that allows the study of the CD4$^+$ T cell response to the dominant H2-A^b-restricted env$_{122–141}$ epitope within the Friend murine leukemia virus (F-MLV) gp70 glycoprotein (Merkenschlager et al., 2016; Thorborn et al., 2014). Small numbers of allotypically marked EF4.1 TCRβ-transgenic CD4$^+$ T cells were transferred into wild-type (WT) C57BL/6 (B6) recipients and primed either by infection with Friend virus (FV) or by immunization with a human Adenovirus 5 (Ad5)-based vector expressing F-MLV gp70 (Ad5.pIX-gp70) (Bayer et al., 2010). FV is a retroviral complex of F-MLV and spleen focus-forming virus (SFFV) that causes chronic infection in B6 mice (Hasenkrug and Chesebro, 1997; Tsuji-Kawahara et al., 2013), whereas the Ad5.pIX-gp70 vaccine vector is replication defective (Bayer et al., 2010). Microarray-based comparison of EF4.1 env-reactive CD4$^+$ T cells primed by Ad5.pIX-gp70 indicated elevated transcription of CTL-related genes, in comparison with T cells primed by FV (Thorborn et al., 2014). Indeed, on day 7 of the response, env-reactive effector CD4$^+$ T cells expressed significantly higher amounts of *Gzmb* mRNA when primed by Ad5.pIX-gp70 than when primed by FV (Figure 1A). Moreover, the hosts exhibited significantly higher levels of MHC class-II-restricted in vivo cytotoxicity against env$_{122–141}$-pulsed B cell targets when primed by Ad5.pIX-gp70 than when primed by FV (Figure 1B). More efficient in vivo killing also correlated with enhanced GzmB-mediated in vitro killing, by purified env-reactive CD4$^+$ T cells, of B cells loaded with a fluorogenic GzmB substrate (Figure 1C).

Consistent with higher *Gzmb* expression and GzmB-mediated killing at the population level, env-reactive effector CD4$^+$ T cells contained a significantly higher proportion of GzmB$^+$ cells if primed by Ad5.pIX-gp70 than if primed by FV (Figure 1D). Notably, GzmB protein expression was detected in env-reactive effector CD4$^+$ T cells even without in vitro restimulation (Figure S1A), suggesting that it reflected in-vivo-induced production. Moreover, EF4.1 env-reactive CD4$^+$ T cells, additionally carrying an allele encoding a fusion of GzmB and tdTomato fluorescent protein (Mouchacca et al., 2013), contained a significantly higher frequency of GzmB-tdTomato$^+$ cells when primed by Ad5.pIX-gp70 than when primed by FV (Figure S1B). Together, these data support the idea that GzmB production was induced in vivo in splenic CD4$^+$ T cells during Ad5.pIX-gp70 immunization. Furthermore, Ad5.pIX-gp70 vaccination induced a significantly higher frequency of GzmB$^+$ cells in splenic host effector CD44$^+$IFN-γ$^+$CD8$^+$ T cells than FV infection did (Figure S2), arguing that the difference between the two immunogens was not restricted to CD4$^+$ T cells or to TCR (T cell-receptor)-transgenic T cells.

One notable difference between FV infection and Ad5.pIX-gp70 immunization is their ability to prime different TCR clonotypes (Thorborn et al., 2014). EF4.1 env-reactive CD4$^+$ T cells induced by FV are primarily TCR Vα2$^+$, whereas those induced by Ad5.pIX-gp70 express a member of the TCR Vα3 family (Thorborn et al., 2014). Differences in TCR usage could underlie the distinct ability of FV and Ad5.pIX-gp70 to induce CD4$^+$ CTLs. Indeed, differentiation of GzmB$^+$ CD4$^+$ T cells was moderately higher in Vα3$^+$ than the Vα2$^+$ fraction of FV-primed env-reactive CD4$^+$ T cells (Figures S3A and S3B). Nevertheless, the two fractions differentiated into GzmB$^+$ CD4$^+$ T cells with comparable efficiency upon Ad5.pIX-gp70 immunization (Figures S3A and S3B). Moreover, Ad5.pIX-gp70 induced significantly stronger *Gzmb* expression in monoclonal TCR-transgenic EVα2 CD4$^+$ T cells than FV infection did (Figure S3C). These results indicated a small effect of TCR usage on CD4$^+$ CTL differentiation, which was, however, overshadowed by other properties of the two viruses.

Lastly, different immunization regimens elicited distinct frequencies of GzmB$^+$ cells within env-reactive effector CD4$^+$ T cells (Figure 1E). These included non-persisting infection with attenuated N-tropic F-MLV (F-MLV-N) (Dittmer et al., 1998) or transient env$_{124–138}$ peptide immunization, which failed to induce GzmB$^+$ cells, and transplantation of the FV-induced FBL-3 tumor cell line (Klarnet et al., 1989), which induced moderate levels of GzmB$^+$ cells (Figure 1E). They also included infection with a replication-competent and persisting mouse-cytomegalovirus (mCMV)-based vector encoding F-MLV *env*, which also induced readily detectable GzmB$^+$ cells (Figure 1E). Thus, properties of the infecting or immunizing virus, independently of its ability to persist in the host, largely determine the efficiency of antigen-specific CD4$^+$ T cell differentiation into CTLs, with Ad5.pIX-gp70 outperforming FV.

Antagonistic CD4$^+$ CTL and Tfh Development

Both TCR usage and the nature of infecting virus can heavily influence Th subset differentiation, which is reflected in the corresponding transcriptional profiles. The necessary cell processing

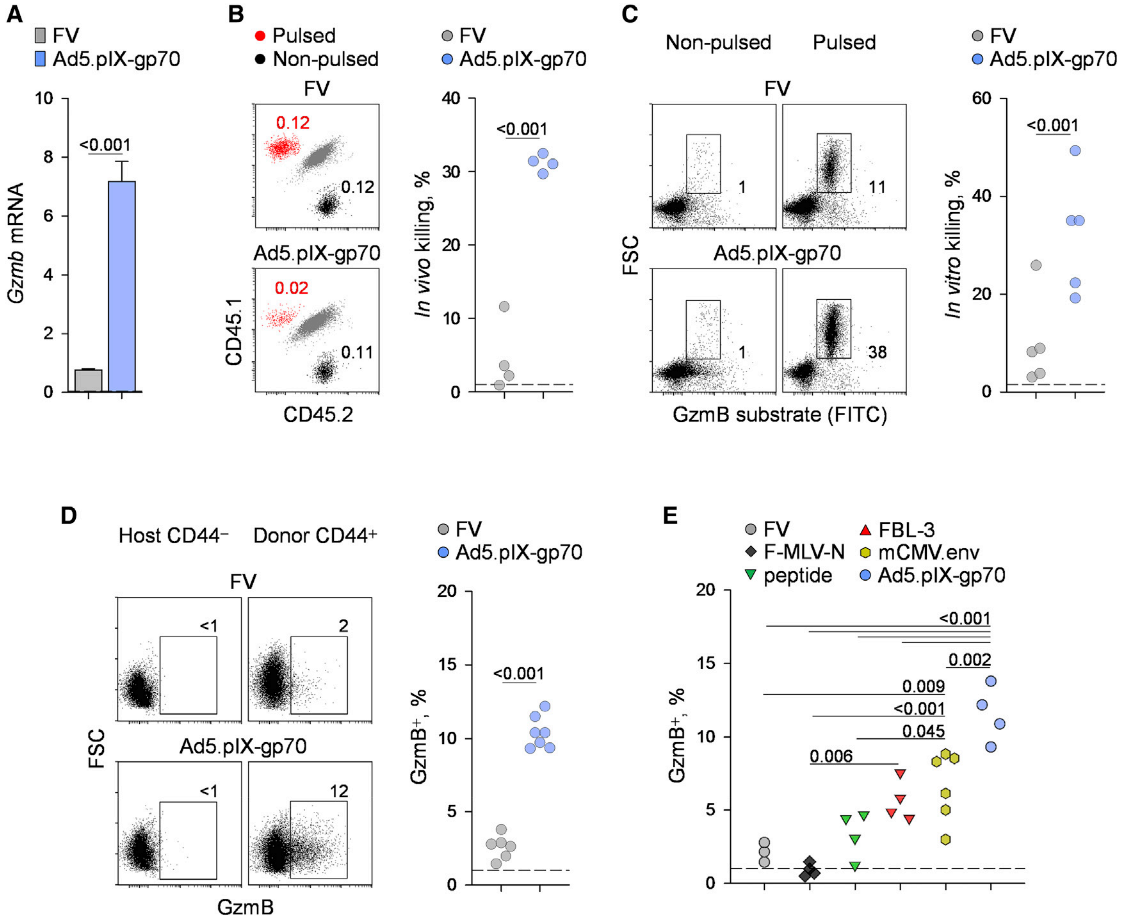

Figure 1. CD4$^+$ CTL Development Depends on Infecting Virus

(A) Expression of *Gzmb*, relative to *Hprt*, assessed by qRT-PCR in env-reactive donor EF4.1 CD4$^+$ T cells purified from the spleens of recipient mice, 7 days after adoptive transfer and FV infection or Ad5.pIX-gp70 immunization. Plotted are the mean values (±SEM) of four technical replicates, from two experiments with five mice per group per experiment.

(B) Flow-cytometric detection (left) and efficiency of in vivo killing (right) of $env_{122-141}$-pulsed CD45.1$^+$ and non-pulsed CD45.2$^+$ B cells in host splenocytes, 24 hr after transfer into CD45.1$^+$CD45.2$^+$ hosts (5×10^6 of each per host) that had also received EF4.1 CD4$^+$ T cells and had either been infected with FV or immunized with Ad5.pIX-gp70 7 days earlier.

(C) Flow-cytometric detection (left) and efficiency of in vitro killing (right) of $env_{122-141}$-pulsed and non-pulsed B cells, 2 hr after culture with env-reactive donor EF4.1 CD4$^+$ T cells purified from the spleens of recipient mice, 7 days after adoptive transfer and FV infection or Ad5.pIX-gp70 immunization.

(D) Flow-cytometric detection of intracellular GzmB host naive (CD44$^-$) or env-reactive (CD44$^+$) donor EF4.1 CD4$^+$ T cells (left) and frequency of GzmB$^+$ cells in env-reactive donor EF4.1 CD4$^+$ T cells (right) in the spleens of recipient mice, 7 days after adoptive transfer and FV infection or Ad5.pIX-gp70 immunization.

(E) Frequency of intracellular GzmB$^+$ cells in env-reactive donor EF4.1 CD4$^+$ T cells in the spleens of recipient mice, 7 days after adoptive transfer and FV infection, F-MLV-N infection, $env_{122-141}$ peptide immunization in the Sigma Adjuvant System, FBL-3 leukemia cell transplantation, mCMV.env infection, or Ad5.pIX-gp70 immunization.

In (B) to (E), each symbol in the scatterplots represents an individual recipient mouse.

See also Figures S1, S2, and S3.

for intracellular GzmB staining precluded further transcriptional analysis between GzmB$^+$ and GzmB$^-$ CD4$^+$ T cells. To overcome this limitation, we performed single-cell RNA sequencing of env-specific CD4$^+$ T cells primed either by FV or Ad5.pIX-gp70. Consistent with flow-cytometric detection of GzmB production, FV induced *Gzmb* expression in 3/57 and 1/65 cells (an average of 3.2%), whereas Ad5.pIX-gp70 induced *Gzmb* expression in 6/42 and 4/45 cells (an average of 11.5%) analyzed in two independent runs ($p = 0.022$, Fisher's exact test) (Figure 2A). In contrast, expression of other cytotoxic mediators, such as *Tnfa*, *Fasl*, and *Tnfsf10*, was comparable between FV and Ad5.pIX-gp70 priming (Figure S4). It should be noted that gene expression assessment by single-cell RNA sequencing represents the lower limit, as it captures only a fraction of the genes expressed in a given cell. This is evident in the transcription of the *Cd4* gene, which is not detected in all of the CD4$^+$ T cells analyzed (Figure 2A).

Single-cell transcriptional analysis revealed another notable difference between FV-primed and Ad5.pIX-gp70-primed CD4$^+$ T cells: a significantly higher proportion of the former transcribed

OPEN ACCESS
CellPress

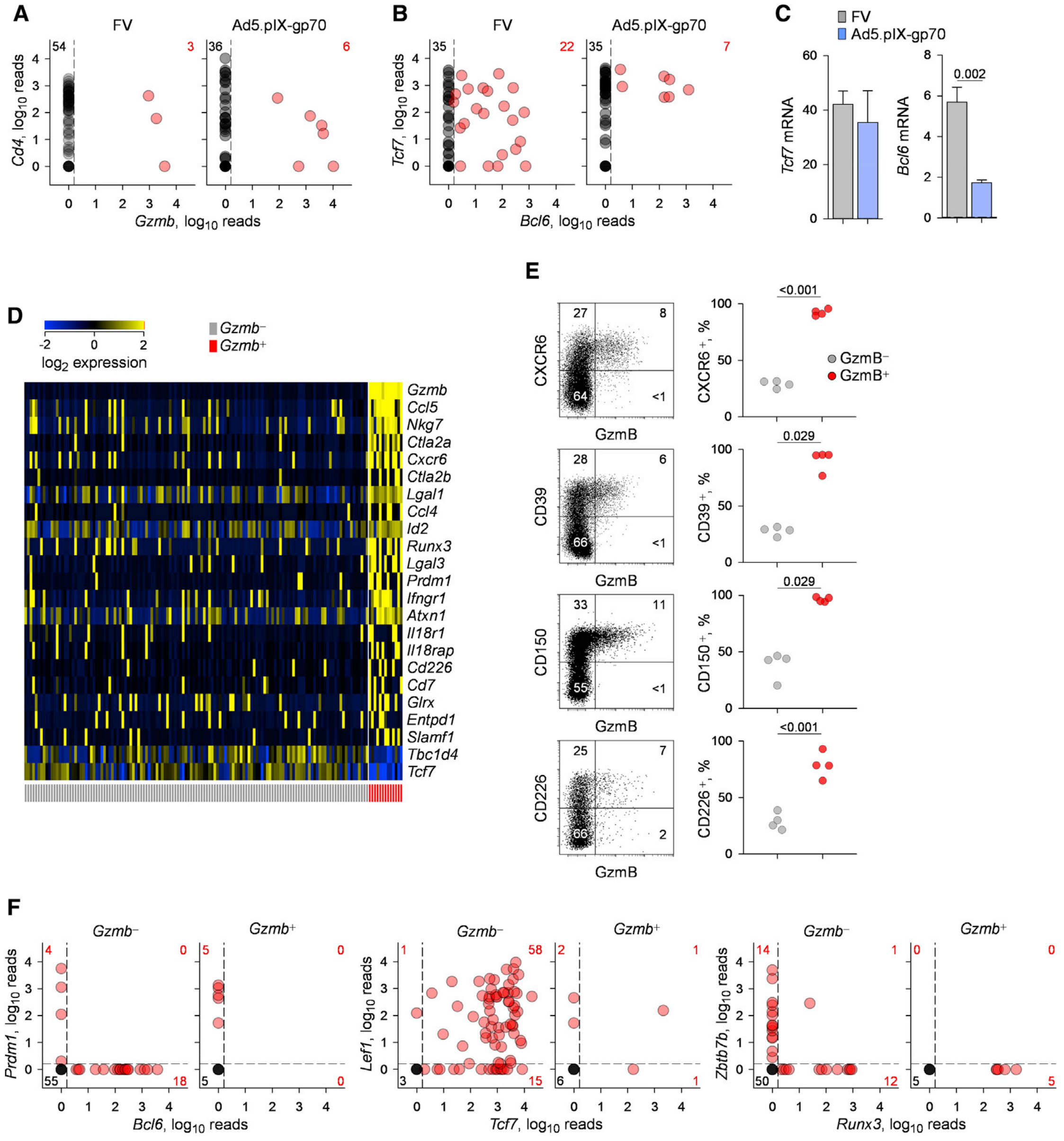

Figure 2. Antagonistic CD4$^+$ CTL and Tfh Development

(A) *Cd4* and *Gzmb* expression, assessed by single-cell RNA sequencing, in env-reactive donor EF4.1 CD4$^+$ T cells purified from the spleens of recipient mice, 7 days after adoptive transfer and FV infection or Ad5.pIX-gp70 immunization. Each symbol shows the $\log_2$-transformed normalized reads from an individual cell from one of two experiments. Numbers within the plots denote the number of cells positive for expression of the indicated gene.

(B) *Tcf7* and *Bcl6* expression in the same cells as in (A).

(C) Expression of *Tcf7* and *Bcl6*, relative to *Hprt*, assessed by qRT-PCR in bulk env-reactive donor EF4.1 CD4$^+$ T cells purified from the spleens of recipient mice, 7 days after adoptive transfer and FV infection or Ad5.pIX-gp70 immunization. Plotted are the mean values (±SEM) of four technical replicates from two experiments with four mice per group per experiment.

(D) Heatmap of gene expression, assessed by single-cell RNA sequencing, comparing *Gzmb*$^+$ and *Gzmb*$^-$ subsets in env-reactive donor EF4.1 CD4$^+$ T cells purified from the spleens of recipient mice, 7 days after adoptive transfer and priming. CD4$^+$ T cells from both FV infection and Ad5.pIX-gp70 immunization are included. A select set of genes from the complete list in Table S1 is shown.

(E) Flow-cytometric correlation of intracellular GzmB and surface markers CXCR6, CD39, CD150, and CD226 (left) and frequency of surface marker$^+$ cells separately in GzmB$^-$ and GzmB$^+$ cells within env-reactive (CD44$^+$) donor EF4.1 CD4$^+$ T cells (right) in the spleens of recipient mice, 7 days after adoptive transfer and Ad5.pIX-gp70 immunization. In the scatterplots, each symbol represents an individual recipient.

(F) Expression of *Prdm1*, *Bcl6*, *Lef1*, *Tcf7*, *Zbtb7b*, and *Runx3* assessed by single-cell RNA sequencing, separately in *Gzmb*$^+$ and *Gzmb*$^-$ env-reactive donor EF4.1 CD4$^+$ T cells purified from the spleens of recipient mice, 7 days after adoptive transfer and Ad5.pIX-gp70 immunization.

See also Figures S4, S5, and S6 and Table S1.

Bcl6 (p = 0.025, Fisher's exact test) (Figure 2B), which is essential for Tfh development (Crotty, 2014; Vinuesa et al., 2016). In contrast, the two types of CD4$^+$ T cells displayed comparable transcription of *Tcf7* (Figure 2B), encoding the transcription factor TCF-1, which has been recently found to promote Tfh development at multiple levels, including through induction of *Bcl6* transcription (Choi et al., 2015; Wu et al., 2015; Xu et al., 2015). Independently assessed in CD4$^+$ T cell populations, levels of *Tcf7* were not significantly lower in Ad5.pIX-gp70-primed than in FV-primed CD4$^+$ T cells, whereas levels of *Bcl6* were (Figure 2C). Together, these results suggested that the degree of CTL and Tfh differentiation in env-specific CD4$^+$ T cells are inversely correlated and dictated by the priming virus.

To examine whether CTL differentiation was inhibited by competing Th programs, we compared the gene transcripts that distinguished *Gzmb*$^+$ cells (Table S1). Interestingly, *Gzmb*$^+$ cells primed by either virus were characterized by specific loss of *Tcf7* expression, among a selected set of genes (Figure 2D). Conversely, *Gzmb*$^+$ cells were characterized by elevated expression of several other genes, including *Cxcr6*, *Entpd1* (encoding CD39), *Slamf1* (encoding CD150), and *Cd226* (Figure 2D), which were further validated by flow cytometry (Figure 2E). These transcriptional differences were also significant when *Gzmb*$^-$ and *Gzmb*$^+$ cells primed by Ad5.pIX-gp70 only were analyzed (Figure S5). Accordingly, none of the Ad5.pIX-gp70-primed *Gzmb*$^+$ cells expressed *Bcl6*, and half of them expressed the antagonistic transcription factor Blimp-1, encoded by *Prdm1*, in sharp contrast to *Gzmb*$^-$ cells (Figure 2F). Also in contrast to *Gzmb*$^-$ cells, which were nearly all *Tcf7*$^+$ and most also expressed *Lef1*, encoding the TCF-1 homolog LEF-1, *Gzmb*$^+$ cells only sporadically expressed *Tcf7* and *Lef1* (Figure 2F; Figure S6A).

The balance of *Zbtb7b* (encoding ThPOK) and *Runx3* transcription, associated with the CD4$^+$ and CD8$^+$ lineages, respectively (Cheroutre and Husain, 2013), was also altered in Ad5.pIX-gp70-primed *Gzmb*$^+$ T cells (Figure 2F; Figure S6A). This observation is consistent with previous reports on intestinal CD4$^+$ CTLs, in which Runx3 expression is associated with CD8α expression (Mucida et al., 2013; Reis et al., 2013). In contrast to intestinal CD4$^+$ CTLs, however, the altered balance of *Zbtb7b* and *Runx3* transcription in splenic *Gzmb*$^+$ CD4$^+$ T cells induced by Ad5.pIX-gp70 did not lead to transcription of either the *Cd8a* or *Cd8b1* genes or the acquisition of *Crtam* or *Eomes* expression (Figure S6B). Lastly, transcription of *Cd5* and *Nr4a1* (encoding Nur77), which could be indicative of the strength of TCR signaling experienced by env-specific CD4$^+$ T cells, did not significantly differ between *Gzmb*$^-$ and *Gzmb*$^+$ cells (Figure S6C), suggesting that TCR signal strength is not the primary determinant of CD4$^+$ CTL differentiation. Collectively, these findings point to a CD4$^+$ CTL-specific transcriptional signature, characterized by acquisition of *Runx3* transcription and, importantly, downregulation of Tfh-related transcription, particularly of *Tcf7*.

Bcl6 Suppresses CD4$^+$ CTL Development

Loss of Tfh-specific transcription in *Gzmb*$^+$ CD4$^+$ T cells suggested that the TCF-1–Bcl6 nexus was incompatible with, or actively inhibiting, CD4$^+$ CTL differentiation. To test this possibility, we used conditional ablation of *Bcl6* in env-specific effector CD4$^+$ T cells, which were transferred into WT hosts (Figure S7). This was achieved by expression of Cre in donor CD4$^+$ T cells under the control of the *Tnfrsf4* promoter (*Tnfrsf4*^{Cre}) (Klinger et al., 2009). This promoter activates in the majority of CD4$^+$ T cells, only following antigen recognition, thus avoiding any effects of gene deletion during T cell development and prior to activation (Marques et al., 2009). Effector CD4$^+$ T cells that activated the *Tnfrsf4* promoter were identified using a Cre-conditional yellow fluorescent protein (YFP) reporter (*Gt(ROSA)26Sor*^{YFP}) allele. A Cre-conditional *Bcl6* (*Bcl6*^{fl}) allele (Kaji et al., 2012) was also introduced in separate donor EF4.1 mice. These combinations created four separate populations of env-specific donor CD4$^+$ T cells (Figure 3A; Figure S7). Particularly, YFP$^-$ CD4$^+$ T cells with (*Bcl6*^{fl}) or without the conditional *Bcl6* allele (*Bcl6*^{wt}) would be comparable, as they retained the capacity to express Bcl6, whereas YFP$^+$ *Bcl6*^{fl}, but not *Bcl6*^{wt}, CD4$^+$ T cells would lose this capacity (Merkenschlager et al., 2016).

Transcriptional analysis of env-specific donor CD4$^+$ T cell populations primed by either virus confirmed significantly higher *Bcl6* expression in FV-primed than in Ad5.pIX-gp70-primed T cells, regardless of *Tnfrsf4* promoter activity (Figure 3B). Moreover, *Bcl6* expression was lost in the YFP$^+$, but not the YFP$^-$, fraction of *Bcl6*^{fl} T cells (Figure 3B), validating the approach. Loss of *Bcl6* expression in the YFP$^+$ fraction was accompanied by significant gain in expression of *Prdm1*, as well as of *Gata3*, *Tbx21*, and *Ifng* (Figure 3B), but not of *Foxp3* or *Rorc* (Figure S8), suggesting that Bcl6 was suppressing the Th1 and Th2 programs. This effect of *Bcl6* deletion on CD4$^+$ T cell differentiation was further confirmed by intracellular staining for T-bet (encoded by *Tbx21*) and interferon (IFN)-γ (Figure S9). Higher levels of T-bet and IFN-γ were induced by FV infection than Ad5.pIX-gp70 immunization in *Bcl6*^{wt} CD4$^+$ T cells, and these were not further elevated in *Bcl6*^{fl} CD4$^+$ T cells (Figure S9). In contrast, the low levels of T-bet and IFN-γ induced by Ad5.pIX-gp70 immunization in *Bcl6*^{wt} CD4$^+$ T cells were significantly elevated in *Bcl6*^{fl} CD4$^+$ T cells (Figure S9).

In addition to enhancing differentiation of other Th cell subsets, loss of *Bcl6* expression led to a striking upregulation of *Gzmb* transcription, specifically in Ad5.pIX-gp70-primed CD4$^+$ T cells (Figure 3B). Importantly, the gain in *Gzmb* expression in the latter population (>13-fold) was considerably more pronounced than the gain in the transcription of the other genes examined (2.2- to 3.8-fold) (Figure 3B). The significantly heightened transcription of *Gzmb*, specifically in Ad5.pIX-gp70 immunization, was additionally confirmed by intracellular staining for GzmB in the total env-specific *Bcl6*^{fl} CD4$^+$ T cell population, containing both Bcl6-deleted and non-deleted cells (Figure 4A).

To gain better insight into the transcriptional profile of only the Bcl6-deleted env-specific CD4$^+$ T cells, without the need for in vitro restimulation, we next subjected the purified YFP$^+$ fraction of *Bcl6*^{fl} CD4$^+$ T cells to single-cell RNA sequencing. This analysis demonstrated a significant increase in the frequency of *Gzmb*$^+$ cells in Bcl6-deleted env-specific CD4$^+$ T cells following priming by Ad5.pIX-gp70 (Figure 4B). Indeed, nearly 40% of these cells were positive for *Gzmb* transcripts (Figure 4B). In contrast, the frequency of *Gzmb*$^+$ cells in Bcl6-deleted env-specific CD4$^+$ T cells primed by FV did not change significantly (Figures 2A and 4B). These data with Bcl6-deleted populations

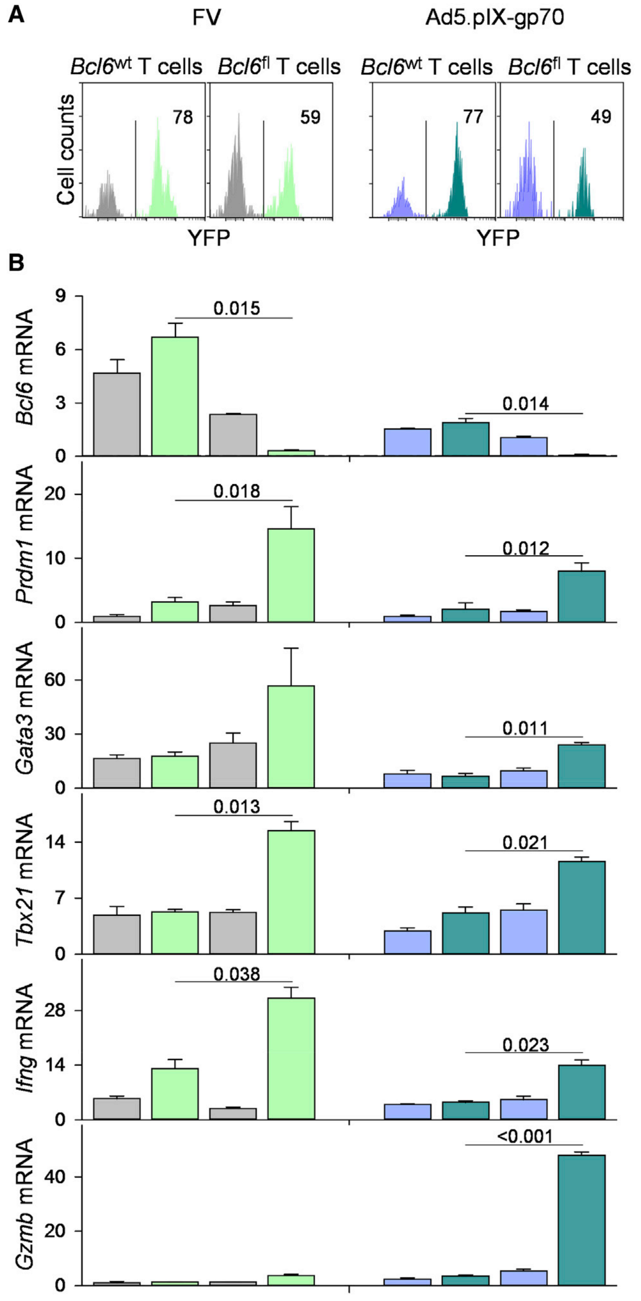

Figure 3. Bcl6 Suppresses CD4$^+$ CTL Development at the Population Level

(A) Delineation of env-reactive donor EF4.1 CD4$^+$ T cells according to YFP expression. Histograms show gated env-reactive EF4.1 CD4$^+$ T cells from *Bcl6*^{wt} or *Bcl6*^{fl} donors, found in the spleens of recipient mice, 7 days after adoptive transfer and FV infection or Ad5.pIX-gp70 immunization. YFP expression reports activation of the *Tnfrsf4* gene and, in the case of *Bcl6*^{fl} donor CD4$^+$ T cells, also loss of *Bcl6*. Numbers within the plots denote the proportion of YFP$^+$ cells.

(B) Expression of the indicated gene, relative to *Hprt*, assessed by qRT-PCR in the respective bulk subset of env-reactive donor EF4.1 CD4$^+$ T cells shown immediately above in (A). Plotted are the mean values (±SEM) of two technical replicates, from two experiments with five mice per group per experiment.

See also Figures S7, S8, and S9.

and purified single cells suggested that Bcl6 was restraining the CTL program in env-specific CD4$^+$ T cells, at least during Ad5.pIX-gp70 priming.

To relate the effect of Bcl6 deletion on *Gzmb* expression, we examined the transcription of additional effector molecules in single WT or Bcl6-deleted env-specific CD4$^+$ T cells. A high proportion (68%) of WT CD4$^+$ T cells primed by FV expressed *Il21* (which, in these settings, was characteristic of the Tfh response), and a smaller proportion (14%) expressed *Il10*, with only partial overlap with *Ifng* expression (Figure 4C). Although loss of *Bcl6* during FV infection did not markedly reduce *Il21* expression, it did significantly enhance expression of both *Ifng* (2.3-fold) and *Il10* (4.7-fold), which were now co-expressed in the majority of *Bcl6*-deleted CD4$^+$ T cells (Figure 4C). In stark contrast to FV infection, Ad5.pIX-gp70 immunization induced very little *Il21* or *Il10* expression in either WT or *Bcl6*-deleted env-specific CD4$^+$ T cells (Figure 4C). Moreover, *Bcl6* deletion induced a more modest gain in *Ifng*-expressing cells (1.5-fold) after Ad5.pIX-gp70 priming than after FV priming (Figure 4C). Collectively, these data argued that Bcl6 suppressed specifically CTL differentiation of env-specific CD4$^+$ T cells during Ad5.pIX-gp70 priming.

CD4$^+$ CTL and Th1 Cells Are Transcriptionally Distinct

GzmB expression is often considered a part of the Th1 program of CD4$^+$ T cell differentiation. Indeed, some of the genes, such as *Slamf1* (encoding CD150), whose expression characterized *Gzmb*$^+$ cells (Figures 2D and 2E), are also used to distinguish Th1 from Tfh cells (Crotty, 2014; Vinuesa et al., 2016). Single-cell transcriptional analysis revealed that, independently of priming virus or Bcl6 sufficiency, over half (57%) of *Gzmb*$^+$ cells co-expressed *Ifng* (Figure 5A). Similar results were obtained at the protein level (Figure 5B), indicating a close relationship between GzmB and IFN-γ production. However, the strong *Ifng* expression in FV infection without concomitant *Gzmb* expression, and the inverse during Ad5.pIX-gp70 immunization, particularly after loss of Bcl6 (Figures 3B, 4A, and 4C), suggested that the profiles of Th1 cells and those that additionally display CTL potential may be separable, both qualitatively and quantitatively. For example, although both Th1 cells and Th1 cells with CTL potential expressed CD150 (encoded by *Slamf1*), a direct comparison of IFN-γ^+ env-specific CD4$^+$ T cells that co-produced GzmB (IFN-γ^+GzmB$^+$) with those that did not (IFN-γ^+GzmB$^-$) revealed significantly higher CD150 expression in the former (Figure 5C).

To comprehensively explore potential transcriptional differences between Th1 and CTL CD4$^+$ T cells, we compared the transcriptional profiles of env-specific CD4$^+$ T cells expressing *Ifng*, but not *Gzmb* (*Ifng*$^+$*Gzmb*$^-$), and those co-expressing *Ifng* and *Gzmb* (*Ifng*$^+$*Gzmb*$^+$). We reasoned that restricting this comparison only to cells expressing *Ifng* would minimize the effect of comparing mixed Th functional subsets and emphasize differences correlating with Gzmb production. Despite common *Ifng* expression, the transcriptional profile of *Ifng*$^+$*Gzmb*$^+$ cells was readily distinguishable from that of *Ifng*$^+$*Gzmb*$^-$ cells (Figure 5D). The transcriptional difference between *Ifng*$^+$*Gzmb*$^+$ and *Ifng*$^+$*Gzmb*$^-$ cells remained significant when Ad5.pIX-gp70 priming was analyzed separately, whereas it narrowly lost significance when FV priming was analyzed in isolation due to the low

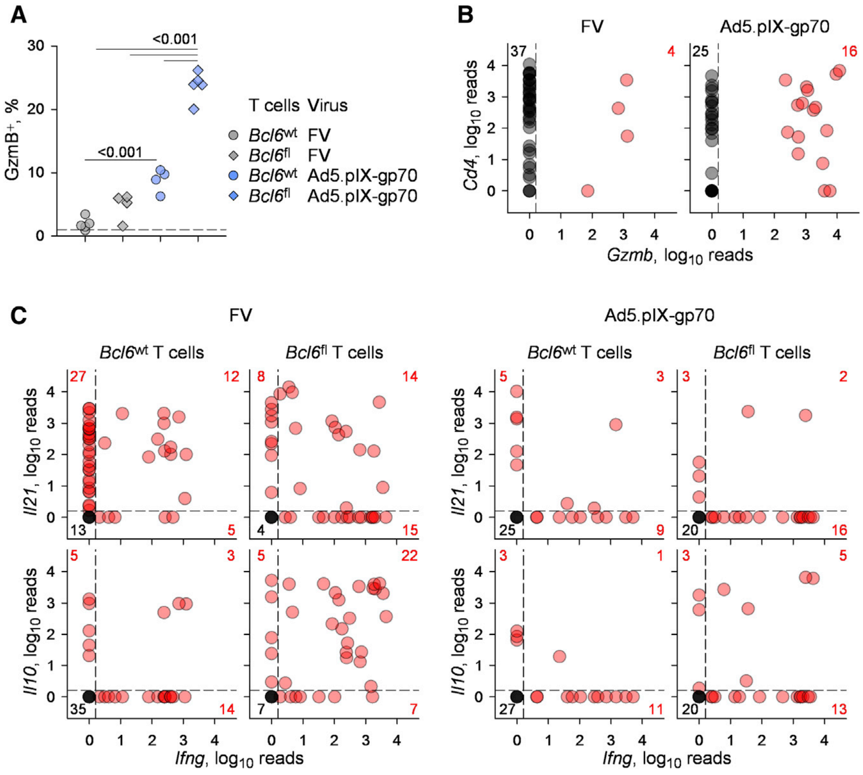

Figure 4. Bcl6 Suppresses CD4$^+$ CTL Development at the Single-Cell Level

(A) Frequency of intracellular GzmB$^+$ cells in bulk *Bcl6*^{wt} or *Bcl6*^{fl} env-reactive donor EF4.1 CD4$^+$ T cells in the spleens of recipient mice, 7 days after adoptive transfer and FV infection or Ad5.pIX-gp70 immunization. Each symbol represents an individual mouse from one representative of two experiments.

(B) *Cd4* and *Gzmb* expression, assessed by single-cell RNA sequencing, in YFP$^+$ *Bcl6*^{fl} env-reactive donor EF4.1 CD4$^+$ T cells purified from the spleens of recipient mice, 7 days after adoptive transfer and FV infection or Ad5.pIX-gp70 immunization.

(C) *Il21*, *Il10*, and *Ifng* expression, assessed by single-cell RNA sequencing, in YFP$^+$ *Bcl6*^{wt}, and *Bcl6*^{fl} env-reactive donor EF4.1 CD4$^+$ T cells purified from the spleens of recipient mice, 7 days after adoptive transfer and FV infection or Ad5.pIX-gp70 immunization.

number of *Gzmb*$^+$ cells induced by FV (Figure S10). Moreover, *Ifng*$^+$*Gzmb*$^+$ and *Ifng*$^+$*Gzmb*$^-$ cells remained transcriptionally significantly distinct when Tfh cells were excluded from the analysis, either by virtue of *Cxcr5* expression or by limiting the analysis to Bcl6-deleted CD4$^+$ T cells, which cannot differentiate into Tfh cells (Figure S11).

Importantly, the transcriptional differences between *Ifng*$^+$ *Gzmb*$^+$ and *Ifng*$^+$*Gzmb*$^-$ cells (Figure 5D) largely overlapped with those characterizing the *Gzmb*$^+$ subset as a whole (Figure 2D). This was particularly evident in the opposing expression of *Prdm1* and *Tcf7*. Indeed, whereas most *Ifng*$^+$*Gzmb*$^-$ cells expressed *Tcf7* but not *Prdm1*, most *Ifng*$^+$*Gzmb*$^+$ cells expressed *Prdm1* but not *Tcf7* (Figure 5E), suggesting that the combination of these two markers was sufficient to distinguish between the Th1 and CD4$^+$ CTLs.

To confirm the distinguishing pattern of *Prdm1* and *Tcf7* expression of *Gzmb*$^+$ CD4$^+$ T cells, we used EF4.1 TCRβ-transgenic CD4$^+$ T cells additionally carrying a GFP reporter into the *Prdm1* locus (Kallies et al., 2009). As GFP insertion disrupts the *Prdm1* gene in these mice (Kallies et al., 2009), we used donors heterozygous for the *Prdm1*^{Gfp} allele to prevent loss of function of the encoded Blimp1 in the adoptively transferred CD4$^+$ T cells. Following Ad5.pIX-gp70 immunization, a small proportion (~8%) of donor env-specific effector CD4$^+$ T cells displayed Blimp1-GFP expression (Figure 5F) and contained *Prdm1* transcripts (Figure 5G). Notably, this fraction was also characterized by paucity of *Tcf7* and overabundance of *Gzmb* transcripts, relative to the Blimp1-GFP$^-$ fraction (Figure 5G). Thus, loss of *Tcf7* expression and induction of *Prdm1* expression could differentiate *Gzmb*$^+$ CD4$^+$ T cells from other Th subsets, including Th1 cells.

Layered Checkpoints in CD4$^+$ CTL Development

Our findings supported a role for Bcl6 in restraining CD4$^+$ CTL development, particularly of CD4$^+$ T cells responding to Ad5.pIX-gp70 immunization (Figures 3 and 4). However, in response to FV infection, *Bcl6* ablation in env-specific CD4$^+$ T cells failed to promote CD4$^+$ CTL differentiation, despite elevated *Prdm1* expression (Figure 3). Therefore, we hypothesized that additional layers of regulation were limiting CD4$^+$ CTL differentiation specifically in FV infection. To test this idea, we compared the expression of inhibitory receptors in env-specific CD4$^+$ T cells primed by either FV or Ad5.pIX-gp70. Following FV infection, the majority (>65%) of *Gzmb*$^-$ cells co-expressed *Pcdc1* (encoding PD-1), *Lag3*, and *Ctla4* but completely lacked expression of *Havcr2* (encoding Tim-3) (Figure 6A). By comparison, following Ad5.pIX-gp70 immunization, *Ctla4* expression was comparable in *Gzmb*$^-$ cells, but expression of *Pcdc1* was reduced to 36%, and expression of *Lag3* was now largely absent (8%) (Figure 6A). Notably, the difference in expression of inhibitory receptors between the two viruses was more pronounced in *Gzmb*$^+$ cells, which, in the case of FV priming, co-expressed all four inhibitory receptors (Figure 6A). In contrast, *Gzmb*$^+$ cells primed by Ad5.pIX-gp70 lacked expression of *Pcdc1* and *Lag3* (Figure 6A). This difference between the two viruses in the induction of *Pcdc1* and *Lag3* expression was also confirmed at the PD-1 and LAG3 protein level. Consistent with the RNA expression data, PD-1 was expressed by nearly all env-specific CD4$^+$ T cells, but at significantly higher levels in FV infection than in Ad5.pIX-gp70 immunization (Figures 6B and 6C). Similarly, a much larger fraction of env-specific CD4$^+$ T cells exhibited LAG3 surface expression when primed by FV than by Ad5.pIX-gp70 (Figure 6B), a difference that was also reflected in the intensity of LAG3 staining of the entire population (Figure 6C). Thus,

OPEN
ACCESS
CellPress

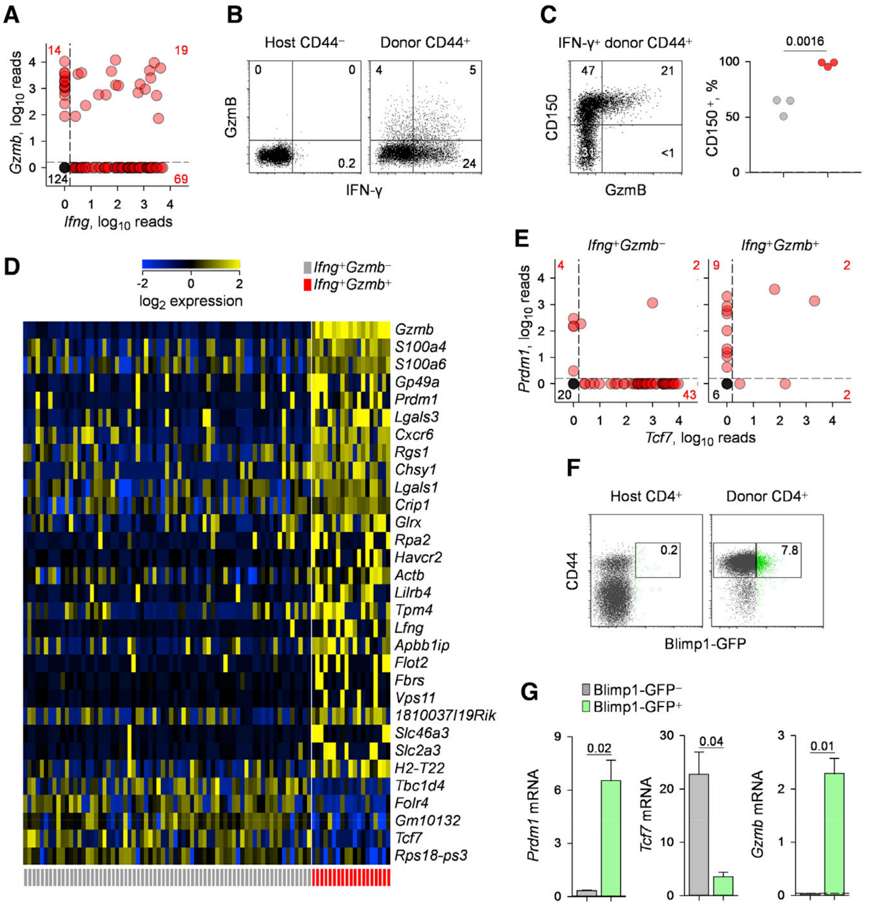

Figure 5. CD4+ CTL and Th1 Cells Are Transcriptionally Distinct

(A) *Gzmb* and *Ifng* expression, assessed by single-cell RNA sequencing, in env-reactive donor EF4.1 CD4+ T cells purified from the spleens of recipient mice, 7 days after adoptive transfer and priming. Both *Bcl6*^{wt} and *Bcl6*^{fl} CD4+ T cells from both FV infection and Ad5.pIX-gp70 immunization are included.

(B) Flow-cytometric detection of intracellular GzmB and IFN-γ in host naive (CD44−) or env-reactive (CD44+) donor EF4.1 CD4+ T cells in the spleens of recipient mice, 7 days after adoptive transfer and Ad5.pIX-gp70 immunization. The plot is representative of four recipients.

(C) Flow-cytometric correlation of intracellular GzmB and surface CD150 expression (left) and frequency of CD150+ cells separately in GzmB− and GzmB+ cells within IFN-γ+ env-reactive (CD44+) donor EF4.1 CD4+ T cells (right) in the spleens of recipient mice, 7 days after adoptive transfer and Ad5.pIX-gp70 immunization. In the scatterplot, each symbol represents an individual recipient.

(D) Heatmap of significantly ($p = 3.95 \times 10^{-4}$) regulated gene expression, assessed by single-cell RNA sequencing, comparing *Ifng*+*Gzmb*+ and *Ifng*+*Gzmb*− subsets in the same cells as in (A).

(E) *Prdm1* and *Tcf7* expression, assessed by single-cell RNA sequencing, in the same cells as in (A).

(F) Flow-cytometric detection of Blimp1-GFP and CD44 expression in host and env-reactive donor CD4+ T cells in the spleens of recipient mice, 7 days after adoptive transfer and Ad5.pIX-gp70 immunization.

(G) Expression of *Prdm1*, *Tcf7*, and *Gzmb*, relative to *Hprt*, assessed by qRT-PCR in bulk Blimp1-GFP+ and Blimp1-GFP− subsets in env-reactive donor EF4.1 CD4+ T cells purified from the spleens of recipient mice, 7 days after adoptive transfer and Ad5.pIX-gp70 immunization. Plotted are the mean values (±SEM) of two technical replicates from one experiment with five mice per group.

See also Figures S10 and S11.

Gzmb+ cells displayed considerable expression of *Ctla4* and exclusive expression of *Havcr2*, regardless of the priming virus. Indeed, *Havcr2* expression also distinguished *Ifng*+*Gzmb*+ cells from *Ifng*+*Gzmb*− cells (Figure 5C). In contrast, significant expression of both *Pcdc1* and *Lag3* was induced in *Gzmb*+ env-specific CD4+ T cells uniquely by FV infection but not Ad5.pIX-gp70 immunization.

The pattern of inhibitory receptors expressed by FV-primed env-specific CD4+ T cells was suggestive of an exhausted phenotype (Crawford et al., 2014), which was investigated further. PD-1 expression was more consistent with antigen-induced activation of effector CD4+ T cells than with cellular exhaustion, as it was also induced by Ad5.pIX-gp70, albeit to a lower intensity per cell (Figure 6C), and was also substantially reduced quickly after the peak of the effector response to FV infection (Figure S12). Moreover, effector CD4+ T cells isolated from acute FV infection were transcriptionally distinct from typical exhausted CD4+ T cells isolated from chronic lymphocytic choriomeningitis virus (LCMV) infection (Crawford et al., 2014; Figure S13A), suggesting that expression of inhibitory receptors by FV-primed effector CD4+ T cells was part of acute effector differentiation rather than of the exhaustion that characterizes chronic viral infections.

Nevertheless, expression of inhibitory receptors, particularly of PD-1 and LAG3, by env-specific CD4+ T cells during acute FV infection could still influence cellular activation or CTL differentiation. To this end, we treated WT recipients of env-specific CD4+ T cells with PD-1- and LAG3-blocking antibodies during the course of FV infection. Although blockade of either PD-1 or LAG3 separately had only a modest effect, the combined PD-1 and LAG3 blockade significantly increased in the frequency of *Gzmb*+ cells in donor CD4+ T cells (Figure 6D; Figure S14), supporting their role in restraining CD4+ CTL differentiation. In contrast, the PD-1 and LAG3 blockade did not appreciably alter clonal expansion of donor CD4+ T cells or their production of IFN-γ and TNF-α (Figure S15).

The high levels of PD-1 expression in env-specific CD4+ T cells responding to FV infection were previously shown to require cognate interaction between T cells and B cells (Ploquin et al., 2011). Therefore, we used B cell deficiency as an alternative to PD-1 blockade. Indeed, a significantly higher proportion of env-specific CD4+ T cells expressed intracellular GzmB when

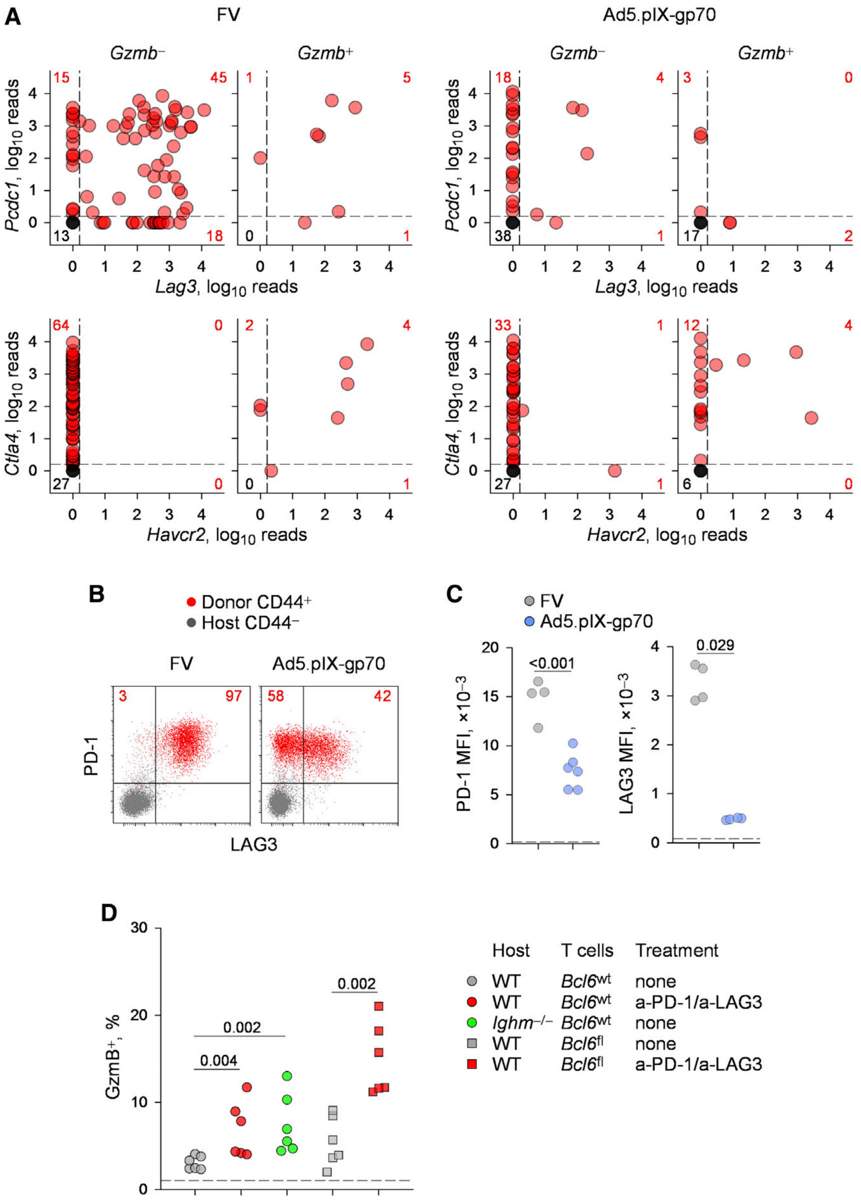

Figure 6. Layered Checkpoints in CD4$^+$ CTL Development

(A) *Pcdc1*, *Lag3*, *Ctla4*, and *Havcr2* expression, assessed by single-cell RNA sequencing, in env-reactive donor EF4.1 CD4$^+$ T cells purified from the spleens of recipient mice, 7 days after adoptive transfer and FV infection or Ad5.pIX-gp70 immunization. Both *Bcl6*^{wt} and *Bcl6*^{fl} CD4$^+$ T cells are included.

(B) Flow-cytometric detection of PD-1 and LAG3 expression in host naive (CD44$^-$) and env-reactive (CD44$^+$) donor CD4$^+$ T cells in the spleens of recipient mice, 7 days after adoptive transfer and FV infection or Ad5.pIX-gp70 immunization. Numbers within the plots denote the proportion of PD-1$^+$ and LAG3$^+$ cells in env-reactive donor CD4$^+$ T cells only.

(C) Median fluorescence intensity (MFI) of PD-1 and LAG3 staining in the same cells as in (B). Each symbol represents an individual recipient. The dashed line represents the MFI of PD-1 and LAG3 staining in host naive CD4$^+$ T cells.

(D) Frequency of intracellular GzmB$^+$ cells in bulk *Bcl6*^{wt} and *Bcl6*^{fl} env-reactive donor EF4.1 CD4$^+$ T cells in the spleens of either WT or B cell-deficient *Ighm*$^{-/-}$ recipient mice, 7 days after adoptive transfer and FV infection. The indicated groups additionally received treatment with PD-1- and LAG3-blocking antibodies. Each symbol represents an individual recipient.

See also Figures S12, S13, S14, and S15.

transferred into B cell-deficient *Ighm*$^{-/-}$ hosts than into WT hosts (Figure 6D). These findings supported the premise that PD-1 and LAG3 posed a further block in CD4$^+$ CTL differentiation, in addition to Bcl6 expression. To test this premise directly, we combined Bcl6 deficiency in env-specific CD4$^+$ T cells with PD-1 and LAG3 blockade. As before (Figure 4A), Bcl6 deficiency alone did not significantly enhance CD4$^+$ CTL differentiation during FV infection (Figure 6D). In contrast, the combination of Bcl6 deficiency and PD-1 and LAG3 blockade markedly increased the proportion of *Gzmb*$^+$ cells (~3-fold) (Figure 6D) to levels comparable with those induced by Bcl6 deficiency in Ad5.pIX-gp70 immunization (Figure 4B). Thus, PD-1 and LAG3 were preventing Bcl6-deficient env-specific CD4$^+$ T cells from acquiring GzmB expression during FV infection, representing an additional level of CD4$^+$ CTL differentiation control.

DISCUSSION

Since the earliest descriptions of MHC class-II-restricted cytotoxic activity in CD4$^+$ T cells nearly 4 decades ago, a number of studies have implicated CD4$^+$ CTLs in antiviral and antitumor immunity, as well as in autoimmune and inflammatory conditions (Brown, 2010; Brown et al., 2016; Cheroutre and Husain, 2013; Soghoian and Streeck, 2010; van de Berg et al., 2008). Nevertheless, the priming requirements for CD4$^+$ CTLs or their phenotypic overlap with other CD4$^+$ Th subsets have only recently begun to emerge. Here, we described the transcriptional profile of CD4$^+$ CTLs as the antipode of the Tfh profile. We provided evidence to suggest multilayered control of CD4$^+$ CTL differentiation: first, by the TCF-1–Bcl6 nexus driving Tfh polarization, and second, inhibition by PD-1 and LAG3.

Study of CD4$^+$ CTLs has been hampered by the lack of distinctive markers that are compatible with further characterization of these cells. Although MHC class-II-restricted cytotoxic activity has been amply documented, it has not been consistently attributed to granzyme-mediated killing, as opposed to killing mediated by secreted or membrane-bound cytokines, including IFN-γ, expressed by Th1 cells, or members of the TNF family, expressed by multiple CD4$^+$ Th cell subsets (Brown et al., 2016; Cheroutre and Husain, 2013). Even when production of GzmB

was used for the identification of CD4$^+$ CTLs, a certain degree of phenotypic overlap with Th1 cells was noted (Hua et al., 2013), and indeed, GzmB-producing CD4$^+$ T cells are still regarded in the literature as a variant of the Th1 subset. This view is further supported by a potential developmental connection between CD4$^+$ CTLs and Th1 cells (Cheroutre and Husain, 2013). Indeed, GzmB-producing cells often also express typical Th1 products, including IFN-γ. CD4$^+$ CTL differentiation, particularly in response to interleukin-2 (IL-2) and IFN-α stimulation, has also been suggested to rely on the Th1-related transcription factor T-bet (encoded by the *Tbx21* gene), which can bind directly to the *Gzmb* promoter (Hua et al., 2013). However, CD4$^+$ CTL differentiation has been reported in other studies to depend not on T-bet but on its homolog Eomesodermin (encoded by the *Eomes* gene) (Hirschhorn-Cymerman et al., 2012; Qui et al., 2011). The differential dependency on either T-bet or Eomesodermin may indicate that, depending on the priming conditions, CD4$^+$ CTLs can develop through separate developmental pathways, which may or may not overlap with the Th1 differentiation pathway. Using a single-cell RNA-sequencing approach, we were able to contrast the entire transcriptome of *Gzmb*$^+$ CD4$^+$ T cells with that of other CD4$^+$ T cells, without prior assumptions of their transcriptional overlap. This approach did not support a correlation between expression of *Gzmb* and expression of either *Tbx21* or *Eomes*, suggesting a certain degree of redundancy. It did, however, reveal a clear distinction between CD4$^+$ T cells with CTL potential and Th1 cells, exemplified in the reciprocal expression of *Tcf7* and *Prdm1*. Although CD4$^+$ CTLs are more similar to the Th1 than other Th subsets, loss of *Tcf7* expression and concomitant gain of *Prdm1* expression in CD4$^+$ CTLs set them apart from all other Th subsets, including Th1 cells. Equally unique was the expression of *Havcr2* (encoding Tim-3), which was found exclusively in CD4$^+$ CTLs.

Although our results uncover prominent transcriptional differences between CD4$^+$ CTLs and Th1 cells, they do not currently inform on any lineage or precursor-product relationship between the two. CD4$^+$ CTLs may represent an advanced or divergent state of Th1 differentiation, characterized by loss of *Tcf7* and acquisition of *Runx3* and *Prdm1* expression. The markers reported here that distinguish CD4$^+$ T cells with CTL potential from Th1 cells will be valuable in determining the transcriptional and phenotypic stability of CD4$^+$ CTLs, or interconversion to a Th1 phenotype, in longitudinal cell-fate studies.

Another defining characteristic of CD4$^+$ CTLs that distinguishes them from other Th subsets is the acquisition of CD8$^+$ lineage-related features. Our analysis confirmed the relative loss of *Zbtb7b* expression (encoding ThPOK) and acquisition of *Runx3* expression, considered responsible for the reprogramming of CD4$^+$ CTLs (Mucida et al., 2013; Reis et al., 2013). However, despite acquiring *Runx3* expression under the conditions we have examined, CD4$^+$ CTLs did not express other CD8$^+$ lineage-related genes, such as *Cd8a* or *Crtam*, previously observed in CD4$^+$ CTLs in other conditions (Mucida et al., 2013; Reis et al., 2013; Takeuchi et al., 2016). It is possible that acquisition of CD8$^+$ lineage-related characteristics is not as extensive in splenic CD4$^+$ CTLs as in intestine intraepithelial CD4$^+$ CTLs. It is also possible that further reprogramming of CD4$^+$ CTLs requires longer antigenic stimulation than the 7 days we have examined here.

Our ability to contrast the global transcriptional profile of *Gzmb*$^+$ CD4$^+$ T cells against that of other CD4$^+$ T cells revealed a principal feature of CD4$^+$ CTL differentiation; namely, antagonism by the Tfh program. Indeed, loss of *Tcf7* and *Lef1* expression distinguished CD4$^+$ CTL from other CD4$^+$ T cell subsets, including *Ifng*$^+$ Th1 cells. TCF-1 (encoded by *Tcf7*) and its homolog, LEF-1, have been recently demonstrated to coordinate Tfh differentiation partly by enhancing *Bcl6* expression during LCMV infection (Choi et al., 2015; Wu et al., 2015; Xu et al., 2015). Interestingly, *Tcf7* and *Lef1* gene deletion in CD4$^+$ T cells in two of these studies promoted transcriptional features of Th1 cells, as well as of CD4$^+$ CTLs, including *Gzmb* expression (Choi et al., 2015; Xu et al., 2015). The acquisition of CTL-related characteristics by *Tcf7*-deficient CD4$^+$ T cells in these population studies was interpreted as part of enhanced Th1 responses (Choi et al., 2015; Xu et al., 2015). Our single-cell analysis clearly demonstrated that the blocking of Tfh differentiation can promote Th1 and CTL differentiation of distinct cells. Not only were Th1 cells and CD4$^+$ CTLs transcriptionally separable, but they were also induced to different degrees in response to different infections. This was exemplified by retroviral infection, where *Bcl6* deficiency promoted Th1, but not CTL, differentiation of CD4$^+$ T cells and adenoviral vaccination, where *Bcl6* deficiency unleashed specifically CTL differentiation.

Antagonism between the Tfh and CD4$^+$ CTL programs is also supported by the contrasting expression of Blimp1 (encoded by *Prdm1*). Blimp1 suppresses both *Bcl6* and *Tcf7* expression and is, in turn, negatively regulated by Bcl6 and TCF-1 (Choi et al., 2015; Wu et al., 2015; Xu et al., 2015). Previous studies demonstrated defective GzmB expression by Blimp1-deficient CD4$^+$ T cells, whereas Blimp1 overexpression enhances CD4$^+$ CTL differentiation (Gong and Malek, 2007; Hua et al., 2013). These studies suggest that CD4$^+$ CTL differentiation requires Blimp1, which is thought to enhance T-bet binding to the promoters of CTL-related genes, including *Gzmb* (Gong and Malek, 2007; Hua et al., 2013). Although Blimp1 expression, in combination with loss of TCF-1, was shown here to be a unique characteristic of CD4$^+$ CTLs, Blimp1 can also be expressed highly by other Th subsets, and its expression alone is not sufficient to drive CD4$^+$ CTL development in our experimental systems. For example, *Bcl6* deficiency induced significantly elevated expression of both *Prdm1* and *Tbx21* in CD4$^+$ T cells responding to FV infection, but it did not enhance CTL differentiation, arguing that Blimp1 may be necessary, but not sufficient, to drive the CTL program in CD4$^+$ T cells.

Retroviral infection was particularly ineffective at inducing CTL differentiation in responding CD4$^+$ T cells. This apparent defect was also observed when Tfh differentiation was precluded by deletion of *Bcl6*, suggesting additional blocks in CD4$^+$ CTL differentiation. Our present findings add the dimension of intrinsic regulation of CD4$^+$ CTL differentiation by the inhibitory receptors PD-1 and LAG3, induced in response, specifically, to retroviral infection. These receptors represented an additional layer of regulation, which prevented *Bcl6*-deficient CD4$^+$ T cells acquiring CTL characteristics. Indeed, CD4$^+$ CTL differentiation in FV infection required the combination of *Bcl6* deletion and PD-1 and LAG3 blockade. In addition to PD-1 and LAG3, CD4$^+$ CTLs were also characterized by expression of the

inhibitory receptor Tim-3 (encoded by *Havcr2*), which was, in fact, entirely restricted to *Gzmb*$^+$ cells. The precise cause of the highly elevated expression of inhibitory receptors in virus-specific effector CD4$^+$ T cells, particularly, in CD4$^+$ CTLs, specifically in response to retroviral infection, remains poorly understood. Notably, GzmB production has been previously detected in CD4$^+$ T cells responding to FV or FV-induced FBL-3 cells, when Treg cells and CD8$^+$ T cells were depleted (Akhmetzyanova et al., 2013; Manzke et al., 2013), suggesting extrinsic regulation. These data imply that both extrinsic regulation, in the form of Treg cells, and intrinsic regulation, in the form of inhibitory receptors in effector T cells, are effectively exploited by retroviruses.

Although incompletely understood, the induction of inhibitory receptors in virus-specific CD4$^+$ T cells during FV infection requires cognate interaction with B cells (Ploquin et al., 2011). Expression of PD-1 in virus-specific CD4$^+$ T cells, for example, is significantly reduced in the absence of B cell antigen presentation during FV infection (Ploquin et al., 2011) or endogenous antigen expression (Han et al., 2010). In keeping with these observations, B cell deficiency also enhanced CD4$^+$ CTL differentiation in this study. These findings are entirely consistent with a critical role for B cells in deciding the balance between Tfh and CTL differentiation of interacting CD4$^+$ T cells. B cells play a well-described role in stabilizing the Tfh program (Crotty, 2014; Vinuesa et al., 2016), and, together with inducing PD-1 expression in CD4$^+$ T cells, they inhibit CD4$^+$ CTL differentiation. Such a role for B cells would ensure efficient antibody production at the expense of CD4$^+$ T cell-mediated immunity. Indeed, B cells inhibit antibody-independent CD4$^+$ T cell-mediated protection against tumors (Qin et al., 1998) or FV-induced erythroblastosis (Pike et al., 2009).

A critical influence of the type of antigen-presenting cell on Tfh and CTL differentiation may also underlie the difference in the efficiency with which distinct viruses or viral vaccines elicit either Tfh or CD4$^+$ CTLs. Elucidation of the role of distinct antigen-presenting cell types in this process may hold the key to both understanding and controlling the balance between Tfh and CD4$^+$ CTLs.

Overall, using single-cell analysis, our study revealed the transcriptional signature of *Gzmb*-expressing CD4$^+$ T cells. Their unique transcriptional features not only support the notion of a distinguishable CD4$^+$ CTL subset but also provide markers for future identification and further longitudinal study of CD4$^+$ CTLs. Additionally, regulation of the CD4$^+$ CTL program by the TCF-1-Bcl6 axis, B cells, and inhibitory receptors, offers a means for manipulating the cytotoxic activity of CD4$^+$ T cells in health and disease.

EXPERIMENTAL PROCEDURES

Mice

Inbred B6 and CD45.1$^+$ congenic B6 (B6.SJL-*Ptprc*^{a} *Pep3*^{b}/BoyJ) mice, TCRβ-transgenic EF4.1 mice (Antunes et al., 2008), TCRαβ doubly transgenic EVα2 mice (Merkenschlager et al., 2016), Rag1-deficient (*Rag1*$^{-/-}$) mice (Mombaerts et al., 1992), B cell-receptor-deficient (*Ighm*$^{-/-}$) mice (Kitamura et al., 1991), mice with an activatable YFP gene targeted into the *Gt(ROSA)26Sor* (*R26*) locus (Srinivas et al., 2001), mice with a targeted insertion of Cre recombinase into the *Tnfrsf4* locus (Klinger et al., 2009) (*Tnfrsf4*^{Cre}), mice with a conditional *Bcl6* allele (Kaji et al., 2012) (*Bcl6*^{fl}), endogenous ecotropic murine-leukemia-virus-deficient (*Emv2*$^{-/-}$) mice (Young et al., 2012), mice with a targeted insertion of GFP into the *Prdm1* locus (Kallies et al., 2009) (Blimp1-GPF), and mice with a targeted insertion of tdTomato fluorescent protein into the *Gzmb* locus (Mouchacca et al., 2013) (GzmB-tdTomato) were all on the B6 genetic background and were maintained at the Francis Crick Institute's animal facilities. All animal experiments were approved by the ethical committee of the Francis Crick Institute and were conducted according to local guidelines and UK Home Office regulations under the Animals (Scientific Procedures Act) 1986 (ASPA).

Retroviral Infection and Immunization

Details of infections, immunizations, and other in vivo treatments can be found in the Supplemental Information.

T Cell Purification, Adoptive Transfer, and Recovery

Single-cell suspensions were prepared from the spleens and lymph nodes of donor CD45.1$^+$ or CD45.2$^+$ EF4.1 mice or CD45.2$^+$ EVα2 mice, and CD4$^+$ T cells were enriched using immunomagnetic positive selection (StemCell Technologies) at >90% purity. A total of 1 × 10^6 EF4.1 CD4$^+$ T cells or 1 × 10^5 EVα2 CD4$^+$ T cells was injected into CD45.1$^+$CD45.2$^+$ recipients via the tail vein. Env-reactive donor CD4$^+$ T cells were purified (>98% purity) from the spleens of recipient mice by cell sorting, performed on MoFlo cell sorters (Dako-Cytomation).

Flow Cytometry

Single-cell suspensions were stained with directly conjugated antibodies to surface markers obtained from eBiosciences, CALTAG/Invitrogen, BD Biosciences, or BioLegend.

For intracellular detection of GzmB, spleen cell suspensions were stimulated for 4 hr with phorbol 14,13 dybutirate (PdBu) and ionomycin (both at 500 ng/ml), in the presence of monensin (2 μg/ml). Cells were then stained for surface antigen and washed; after this step, they were fixed and permeabilized using an anti-mouse/rat FoxP3 staining kit (eBioscience), according to the manufacturer's instructions. After an additional wash step, cells were stained for intracellular GzmB with Alexa Fluor 647- or FITC (fluorescein isothiocyanate)-conjugated anti-human/mouse GzmB antibodies (clone GB11, Biolegend) and phycoerythrin (PE)-conjugated anti-mouse IFN-γ antibodies (clone XMG1.2, eBioscience). Multi-color cytometry was performed on Canto II or LSRFortessa X-20 flow cytometers (both from BD Biosciences) and analyzed with FlowJo v10 (Tree Star).

Cytotoxicity Assays

Details of in vitro and in vivo cytotoxicity assays can be found in the Supplemental Information.

PCR-Based Expression Profiling

Expression of selected genes was quantified in env-reactive CD4$^+$ T cells by real-time qRT-PCR. The indicated CD4$^+$ T cell populations were purified by cell sorting, and RNA was isolated using the QIAcube (QIAGEN). Synthesis of cDNA was carried out with the High Capacity Reverse Transcription Kit (Applied Biosystems) with an added RNase inhibitor (Promega Biosciences). A final clean-up was performed with the QIAquick PCR Purification Kit (QIAGEN). Purified cDNA was then used as template for the quantitation of the indicated genes using gene-specific primers (Eurofins MWG Operon) (Table S2). Values were normalized and plotted according to the expression of *Hprt* in the same samples, using a ΔC_T method.

Single-Cell RNA Sequencing

Env-reactive CD4$^+$ T cells from the indicated recipient mice were purified by cell sorting. A detailed description of subsequent single-cell RNA sequencing can be found in the Supplemental Information.

Statistical Analyses

Statistical comparisons were made using SigmaPlot 12.0 (Systat Software). Parametric comparisons of normally distributed values that satisfied the variance criteria were made by unpaired Student's t tests or one-way ANOVAs.

Data that did not pass the variance test were compared with non-parametric two-tailed Mann-Whitney rank sum tests or ANOVA on ranks tests. Hierarchical clustering and heatmap production was performed with Qlucore Omics Explorer (Qlucore).

ACCESSION NUMBERS

The accession number for the single-cell RNA sequences reported in this paper is ENA: PRJEB14043.

SUPPLEMENTAL INFORMATION

Supplemental Information includes Supplemental Experimental Procedures, 15 figures, and two tables and can be found with this article online at http://dx.doi.org/10.1016/j.celrep.2016.10.013.

AUTHOR CONTRIBUTIONS

Conceptualization, G.K.; Methodology and Formal Analysis, G.R.Y.; Investigation, T.D., J.M., U.E., and N.B.; Resources, W.B., U.D., N.B., V.T.K.L-T., M.T., S.L.N., and C.B.; Funding Acquisition, G.K.; Supervision, G.K.; Writing – Original Draft, T.D., G.R.Y., and G.K.; Writing – Review & Editing, G.K.

ACKNOWLEDGMENTS

We wish to thank Dr. Toshitada Takemori for the conditional Bcl6 mice. We are grateful for assistance from the Flow Cytometry and Advanced Sequencing Facilities at the Francis Crick Institute. This work was supported by the Francis Crick Institute, which receives its core funding from Cancer Research UK (FC001099), the UK Medical Research Council (FC001099), and the Wellcome Trust (FC001099); and by the Deutsche Forschungsgemeinschaft (DFG-TRR60 and DFG-GRK 1949).

Received: June 7, 2016
Revised: August 31, 2016
Accepted: October 4, 2016
Published: November 1, 2016

REFERENCES

Akhmetzyanova, I., Zelinskyy, G., Schimmer, S., Brandau, S., Altenhoff, P., Sparwasser, T., and Dittmer, U. (2013). Tumor-specific CD4+ T cells develop cytotoxic activity and eliminate virus-induced tumor cells in the absence of regulatory T cells. Cancer Immunol. Immunother. *62*, 257–271.

Antunes, I., Tolaini, M., Kissenpfennig, A., Iwashiro, M., Kuribayashi, K., Malissen, B., Hasenkrug, K., and Kassiotis, G. (2008). Retrovirus-specificity of regulatory T cells is neither present nor required in preventing retrovirus-induced bone marrow immune pathology. Immunity *29*, 782–794.

Bayer, W., Tenbusch, M., Lietz, R., Johrden, L., Schimmer, S., Uberla, K., Dittmer, U., and Wildner, O. (2010). Vaccination with an adenoviral vector that encodes and displays a retroviral antigen induces improved neutralizing antibody and CD4+ T-cell responses and confers enhanced protection. J. Virol. *84*, 1967–1976.

Brown, D.M. (2010). Cytolytic CD4 cells: direct mediators in infectious disease and malignancy. Cell. Immunol. *262*, 89–95.

Brown, D.M., Lampe, A.T., and Workman, A.M. (2016). The differentiation and protective function of cytolytic CD4 T cells in influenza infection. Front. Immunol. *7*, 93.

Cheroutre, H., and Husain, M.M. (2013). CD4 CTL: living up to the challenge. Semin. Immunol. *25*, 273–281.

Choi, Y.S., Gullicksrud, J.A., Xing, S., Zeng, Z., Shan, Q., Li, F., Love, P.E., Peng, W., Xue, H.H., and Crotty, S. (2015). LEF-1 and TCF-1 orchestrate T(FH) differentiation by regulating differentiation circuits upstream of the transcriptional repressor Bcl6. Nat. Immunol. *16*, 980–990.

Crawford, A., Angelosanto, J.M., Kao, C., Doering, T.A., Odorizzi, P.M., Barnett, B.E., and Wherry, E.J. (2014). Molecular and transcriptional basis of $CD4^+$ T cell dysfunction during chronic infection. Immunity *40*, 289–302.

Crotty, S. (2014). T follicular helper cell differentiation, function, and roles in disease. Immunity *41*, 529–542.

Cullen, S.P., Brunet, M., and Martin, S.J. (2010). Granzymes in cancer and immunity. Cell Death Differ. *17*, 616–623.

Dittmer, U., Brooks, D.M., and Hasenkrug, K.J. (1998). Characterization of a live-attenuated retroviral vaccine demonstrates protection via immune mechanisms. J. Virol. *72*, 6554–6558.

Gong, D., and Malek, T.R. (2007). Cytokine-dependent Blimp-1 expression in activated T cells inhibits IL-2 production. J. Immunol. *178*, 242–252.

Han, S., Asoyan, A., Rabenstein, H., Nakano, N., and Obst, R. (2010). Role of antigen persistence and dose for CD4+ T-cell exhaustion and recovery. Proc. Natl. Acad. Sci. USA *107*, 20453–20458.

Hasenkrug, K.J., and Chesebro, B. (1997). Immunity to retroviral infection: the Friend virus model. Proc. Natl. Acad. Sci. USA *94*, 7811–7816.

Hirschhorn-Cymerman, D., Budhu, S., Kitano, S., Liu, C., Zhao, F., Zhong, H., Lesokhin, A.M., Avogadri-Connors, F., Yuan, J., Li, Y., et al. (2012). Induction of tumoricidal function in CD4+ T cells is associated with concomitant memory and terminally differentiated phenotype. J. Exp. Med. *209*, 2113–2126.

Hua, L., Yao, S., Pham, D., Jiang, L., Wright, J., Sawant, D., Dent, A.L., Braciale, T.J., Kaplan, M.H., and Sun, J. (2013). Cytokine-dependent induction of CD4+ T cells with cytotoxic potential during influenza virus infection. J. Virol. *87*, 11884–11893.

Kaji, T., Ishige, A., Hikida, M., Taka, J., Hijikata, A., Kubo, M., Nagashima, T., Takahashi, Y., Kurosaki, T., Okada, M., et al. (2012). Distinct cellular pathways select germline-encoded and somatically mutated antibodies into immunological memory. J. Exp. Med. *209*, 2079–2097.

Kallies, A., Xin, A., Belz, G.T., and Nutt, S.L. (2009). Blimp-1 transcription factor is required for the differentiation of effector CD8(+) T cells and memory responses. Immunity *31*, 283–295.

Kitamura, D., Roes, J., Kühn, R., and Rajewsky, K. (1991). A B cell-deficient mouse by targeted disruption of the membrane exon of the immunoglobulin mu chain gene. Nature *350*, 423–426.

Klarnet, J.P., Kern, D.E., Okuno, K., Holt, C., Lilly, F., and Greenberg, P.D. (1989). FBL-reactive CD8+ cytotoxic and CD4+ helper T lymphocytes recognize distinct Friend murine leukemia virus-encoded antigens. J. Exp. Med. *169*, 457–467.

Klinger, M., Kim, J.K., Chmura, S.A., Barczak, A., Erle, D.J., and Killeen, N. (2009). Thymic OX40 expression discriminates cells undergoing strong responses to selection ligands. J. Immunol. *182*, 4581–4589.

Manzke, N., Akhmetzyanova, I., Hasenkrug, K.J., Trilling, M., Zelinskyy, G., and Dittmer, U. (2013). CD4+ T cells develop antiretroviral cytotoxic activity in the absence of regulatory T cells and CD8+ T cells. J. Virol. *87*, 6306–6313.

Marques, R., Williams, A., Eksmond, U., Wullaert, A., Killeen, N., Pasparakis, M., Kioussis, D., and Kassiotis, G. (2009). Generalized immune activation as a direct result of activated CD4+ T cell killing. J. Biol. *8*, 93.

Merkenschlager, J., Ploquin, M.J., Eksmond, U., Andargachew, R., Thorborn, G., Filby, A., Pepper, M., Evavold, B., and Kassiotis, G. (2016). Stepwise B-cell-dependent expansion of T helper clonotypes diversifies the T-cell response. Nat. Commun. *7*, 10281.

Mombaerts, P., Iacomini, J., Johnson, R.S., Herrup, K., Tonegawa, S., and Papaioannou, V.E. (1992). RAG-1-deficient mice have no mature B and T lymphocytes. Cell *68*, 869–877.

Mouchacca, P., Schmitt-Verhulst, A.M., and Boyer, C. (2013). Visualization of cytolytic T cell differentiation and granule exocytosis with T cells from mice expressing active fluorescent granzyme B. PLoS ONE *8*, e67239.

Mucida, D., Husain, M.M., Muroi, S., van Wijk, F., Shinnakasu, R., Naoe, Y., Reis, B.S., Huang, Y., Lambolez, F., Docherty, M., et al. (2013). Transcriptional reprogramming of mature $CD4^+$ helper T cells generates distinct MHC class II-restricted cytotoxic T lymphocytes. Nat. Immunol. *14*, 281–289.

O'Shea, J.J., and Paul, W.E. (2010). Mechanisms underlying lineage commitment and plasticity of helper CD4+ T cells. Science *327*, 1098–1102.

Pike, R., Filby, A., Ploquin, M.J., Eksmond, U., Marques, R., Antunes, I., Hasenkrug, K., and Kassiotis, G. (2009). Race between retroviral spread and CD4+ T-cell response determines the outcome of acute Friend virus infection. J. Virol. *83*, 11211–11222.

Ploquin, M.J., Eksmond, U., and Kassiotis, G. (2011). B cells and TCR avidity determine distinct functions of CD4+ T cells in retroviral infection. J. Immunol. *187*, 3321–3330.

Qin, Z., Richter, G., Schüler, T., Ibe, S., Cao, X., and Blankenstein, T. (1998). B cells inhibit induction of T cell-dependent tumor immunity. Nat. Med. *4*, 627–630.

Qui, H.Z., Hagymasi, A.T., Bandyopadhyay, S., St. Rose, M.C., Ramanarasimhaiah, R., Ménoret, A., Mittler, R.S., Gordon, S.M., Reiner, S.L., Vella, A.T., and Adler, A.J. (2011). CD134 plus CD137 dual costimulation induces Eomesodermin in CD4 T cells to program cytotoxic Th1 differentiation. J. Immunol. *187*, 3555–3564.

Reis, B.S., Rogoz, A., Costa-Pinto, F.A., Taniuchi, I., and Mucida, D. (2013). Mutual expression of the transcription factors Runx3 and ThPOK regulates intestinal $CD4^+$ T cell immunity. Nat. Immunol. *14*, 271–280.

Soghoian, D.Z., and Streeck, H. (2010). Cytolytic CD4(+) T cells in viral immunity. Expert Rev. Vaccines *9*, 1453–1463.

Srinivas, S., Watanabe, T., Lin, C.S., William, C.M., Tanabe, Y., Jessell, T.M., and Costantini, F. (2001). Cre reporter strains produced by targeted insertion of EYFP and ECFP into the ROSA26 locus. BMC Dev. Biol. *1*, 4.

Swain, S.L., McKinstry, K.K., and Strutt, T.M. (2012). Expanding roles for $CD4^+$ T cells in immunity to viruses. Nat. Rev. Immunol. *12*, 136–148.

Takeuchi, A., Badr Mel, S., Miyauchi, K., Ishihara, C., Onishi, R., Guo, Z., Sasaki, Y., Ike, H., Takumi, A., Tsuji, N.M., et al. (2016). CRTAM determines the CD4+ cytotoxic T lymphocyte lineage. J. Exp. Med. *213*, 123–138.

Thorborn, G., Ploquin, M.J., Eksmond, U., Pike, R., Bayer, W., Dittmer, U., Hasenkrug, K.J., Pepper, M., and Kassiotis, G. (2014). Clonotypic composition of the CD4+ T cell response to a vectored retroviral antigen is determined by its speed. J. Immunol. *193*, 1567–1577.

Tsuji-Kawahara, S., Kawabata, H., Matsukuma, H., Kinoshita, S., Chikaishi, T., Sakamoto, M., Kawasaki, Y., and Miyazawa, M. (2013). Differential requirements of cellular and humoral immune responses for Fv2-associated resistance to erythroleukemia and for regulation of retrovirus-induced myeloid leukemia development. J. Virol. *87*, 13760–13774.

van de Berg, P.J., van Leeuwen, E.M., ten Berge, I.J., and van Lier, R. (2008). Cytotoxic human CD4(+) T cells. Curr. Opin. Immunol. *20*, 339–343.

Vinuesa, C.G., Linterman, M.A., Yu, D., and MacLennan, I.C. (2016). Follicular helper T cells. Annu. Rev. Immunol. *34*, 335–368.

Wu, T., Shin, H.M., Moseman, E.A., Ji, Y., Huang, B., Harly, C., Sen, J.M., Berg, L.J., Gattinoni, L., McGavern, D.B., and Schwartzberg, P.L. (2015). TCF1 is required for the T follicular helper cell response to viral infection. Cell Rep. *12*, 2099–2110.

Xu, L., Cao, Y., Xie, Z., Huang, Q., Bai, Q., Yang, X., He, R., Hao, Y., Wang, H., Zhao, T., et al. (2015). The transcription factor TCF-1 initiates the differentiation of T(FH) cells during acute viral infection. Nat. Immunol. *16*, 991–999.

Young, G.R., Ploquin, M.J., Eksmond, U., Wadwa, M., Stoye, J.P., and Kassiotis, G. (2012). Negative selection by an endogenous retrovirus promotes a higher-avidity CD4+ T cell response to retroviral infection. PLoS Pathog. *8*, e1002709.

Zhu, J., Yamane, H., and Paul, W.E. (2010). Differentiation of effector CD4 T cell populations (*). Annu. Rev. Immunol. *28*, 445–489.

Cell Reports, Volume *17*

Supplemental Information

Opposing Development of Cytotoxic and Follicular Helper CD4 T Cells Controlled by the TCF-1-Bcl6 Nexus

Tiziano Donnarumma, George R. Young, Julia Merkenschlager, Urszula Eksmond, Nadine Bongard, Stephen L. Nutt, Claude Boyer, Ulf Dittmer, Vu Thuy Khanh Le-Trilling, Mirko Trilling, Wibke Bayer, and George Kassiotis

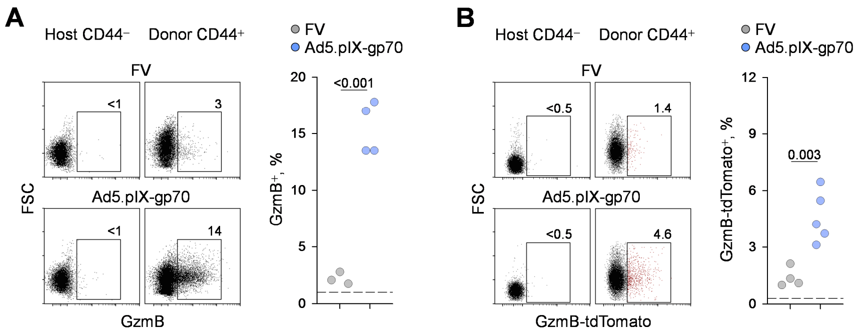

Figure S1. Related to Figure 1; Detection of GzmB production without *in vitro* restimulation

(A) Flow cytometric detection of intracellular GzmB in host naïve ($CD44^-$) or env-reactive ($CD44^+$) donor EF4.1 $CD4^+$ T cells (*left*) and frequency of $GzmB^+$ cells in env-reactive donor EF4.1 $CD4^+$ T cells (*right*) in the spleens of recipient mice, 7 days after adoptive transfer and FV infection or Ad5.pIX-gp70 immunization, without an *in vitro* restimulation step. In the scatter plot, each symbol is an individual recipient. One representative of two experiments is shown.

(B) Flow cytometric detection of tdTomato fluorescence from a chimeric GzmB protein in host naïve ($CD44^-$) or env-reactive ($CD44^+$) donor *Gzmb*^{tdTom} EF4.1 $CD4^+$ T cells (*left*) and frequency of GzmB-$tdTomato^+$ cells in env-reactive donor *Gzmb*^{tdTom} EF4.1 $CD4^+$ T cells (*right*) in the spleens of recipient mice, 7 days after adoptive transfer and FV infection or Ad5.pIX-gp70 immunization. In the scatter plot, each symbol is an individual recipient from a single experiment.

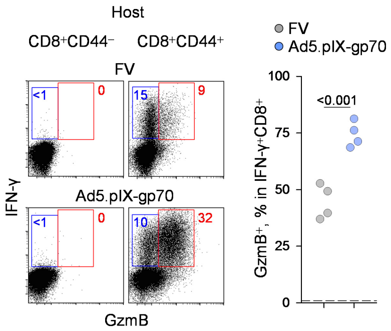

Figure S2. Related to Figure 1; CD8$^+$ CTL development depends on infecting virus

(A) Flow cytometric detection of intracellular GzmB and IFN-γ in naïve (CD44$^-$) or memory-phenotype (CD44$^+$) host CD8$^+$ T cells (*left*) and frequency of GzmB$^+$ cells in CD44$^+$IFN-γ$^+$CD8$^+$ T cells (*right*) in the spleens of mice, 7 days after FV infection or Ad5.pIX-gp70 immunization. In the scatter plot, each symbol is an individual recipient. One representative of three experiments is shown.

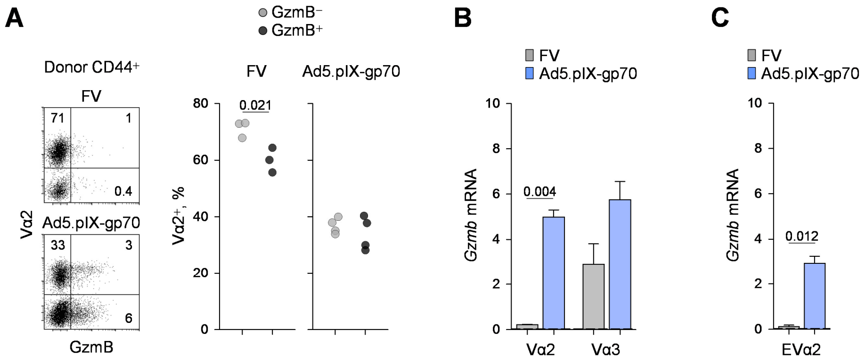

Figure S3. Related to Figure 1; Effect of TCR avidity on CD4⁺ CTL differentiation

(A) Flow cytometric detection of intracellular GzmB according to TCR Vα2 expression in env-reactive (CD44⁺) donor EF4.1 CD4⁺ T cells (*left*) and frequency of Vα2⁺ cells in the GzmB⁻ and GzmB⁺ subsets of env-reactive donor EF4.1 CD4⁺ T cells in the spleens of recipient mice, 7 days after adoptive transfer and FV infection or Ad5.pIX-gp70 immunization. In the scatter plot, each symbol is an individual recipient.

(B) Expression of *Gzmb*, relative to *Hprt*, assessed by qRT-PCR in the Vα2 and Vα3 subsets of env-reactive donor EF4.1 CD4⁺ T cells purified from the spleens of recipient mice, 7 days after adoptive transfer and FV infection or Ad5.pIX-gp70 immunization. Plotted are the mean values (±SEM) of 2 technical replicates, from 2 experiments with 4 mice per group per experiment.

(C) Expression of *Gzmb*, relative to *Hprt*, assessed by qRT-PCR in env-reactive donor monoclonal EVα2 CD4⁺ T cells purified from the spleens of recipient mice, 7 days after adoptive transfer and FV infection or Ad5.pIX-gp70 immunization. Plotted are the mean values (±SEM) of 2 technical replicates, from 1 experiment with 5 mice per group.

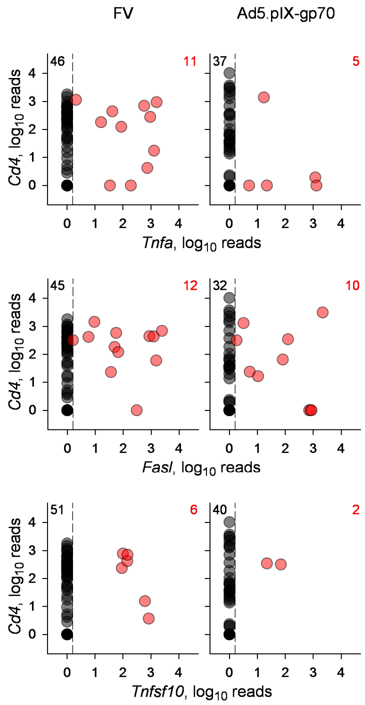

Figure S4. Related to Figure 2; Comparable *Tnfa*, *Fasl* and *Tnfsf10* induction in CD4^{+} CTLs by FV infection and Ad5.pIX-gp70 immunization

Expression of *Tnfa, Fasl and Tnfsf10*, assessed by single-cell RNA sequencing, in env-reactive donor EF4.1 CD4^{+} T cells purified from the spleens of recipient mice, 7 days after adoptive transfer and FV infection or Ad5.pIX-gp70 immunization.

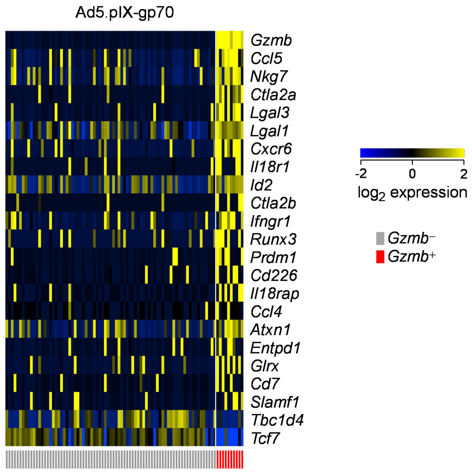

Figure S5. Related to Figure 2; Transcriptional signature of CD4$^+$ CTLs induced by Ad5.pIX-gp70 immunization

Heat-map of gene expression, assessed by single-cell RNA sequencing, comparing *Gzmb*$^+$ and *Gzmb*$^-$ subsets in env-reactive donor EF4.1 CD4$^+$ T cells purified from the spleens of recipient mice, 7 days after adoptive transfer and priming. CD4$^+$ T cells only from Ad5.pIX-gp70 immunization are included. Numbers of *Gzmb*$^+$ cells induced by FV infection were too low to allow for a similar comparison.

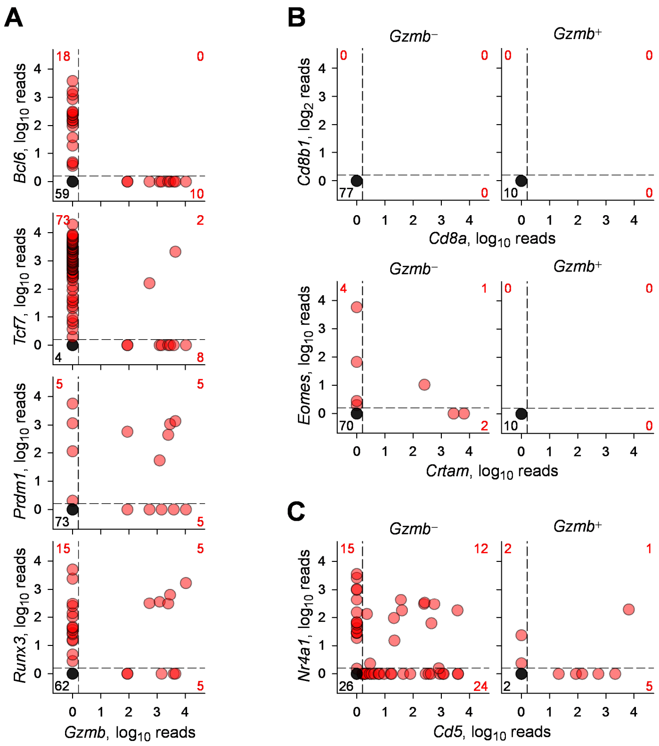

Figure S6. Related to Figure 2; Transcriptional comparison of single *Gzmb*$^{+}$ and *Gzmb*$^{-}$ CD4^{+} T cells

(A) Expression of *Bcl6*, *Tcf7*, *Prdm1* and *Runx3*, according to *Gzmb* expression, assessed by single-cell RNA sequencing, in env-reactive donor EF4.1 CD4^{+} T cells purified from the spleens of recipient mice, 7 days after adoptive transfer and Ad5.pIX-gp70 immunization.

(B) Expression of *Cd8a* and *Cd8b1* (*top*) and *Crtam and Eomes* (*bottom*), assessed by single-cell RNA sequencing, separately in *Gzmb*$^{+}$ and *Gzmb*$^{-}$ env-reactive donor EF4.1 CD4^{+} T cells purified from the spleens of recipient mice, 7 days after adoptive transfer and Ad5.pIX-gp70 immunization.

(C) Expression of *Cd5* and *Nr4a1* in the same cells as in B.

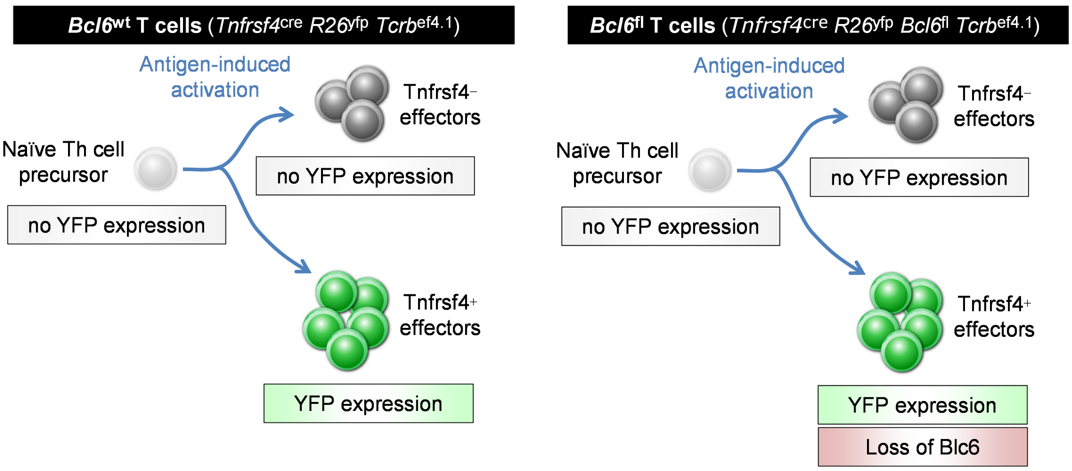

Figure S7. Related to Figure 3; Strategy for conditional deletion of *Bcl6* in effector CD4$^+$ T cells

Conditional ablation of *Bcl6* in env-specific effector CD4$^+$ T cells is achieved by expression of Cre in donor CD4$^+$ T cells, transferred into wild-type recipients, under the control of the *Tnfrsf4* promoter (*Tnfrsf4*^{Cre}). Cells that activated Cre are identified by the use of a conditional YFP reporter (*Gt(ROSA)26Sor*^{YFP}). Naïve Th cell precursors, which do not express *Tnfrsf4*, are unaffected and they neither gain YFP nor lose Bcl6.

In control *Bcl6*^{wt} mice (*left*), antigen-induced activation of env-specific naïve Th cell precursors leads to *Tnfrsf4* transcription in a proportion (70-80%) of cells. In turn, this drives *Cre* transcription, ultimately leading to YFP expression. In these mice, YFP marks the cells that activate the *Tnfrsf4* promoter.

In *Bcl6*^{fl} mice (*right*), additionally carrying the Cre-conditional *Bcl6*^{fl} allele, antigen-induced activation of env-specific naïve Th cell precursors leads not only to YFP expression, but also to loss of Bcl6 in the same cells.

The YFP-expressing populations between the two types of donors represent CD4$^+$ T cells at the same state of activation (but differing in Bcl6 expression) and are directly comparable.

Both types of donor CD4$^+$ T cells also contain YFP-negative effector cells that do not activate the *Tnfrsf4* promoter and therefore do not lose the capacity to express Bcl6. These YFP-negative populations additionally serve as internal controls.

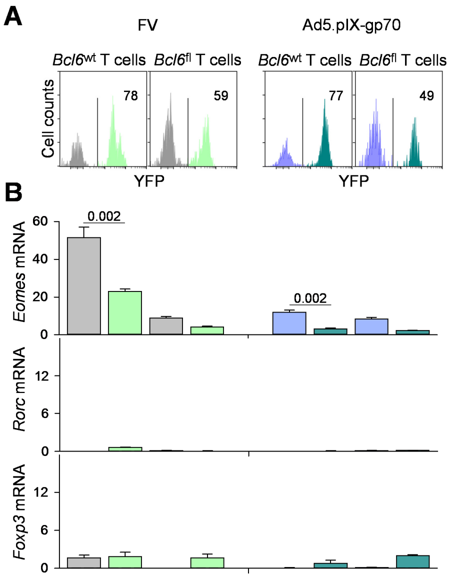

Figure S8. Related to Figure 3; Bcl6 suppresses CD4^{+} CTL development

(A) Delineation of env-reactive donor EF4.1 CD4^{+} T cells according to YFP expression as in Fig. S7.

(B) Expression of the indicated gene, relative to *Hprt*, assessed by qRT-PCR in the respective bulk subset of env-reactive donor EF4.1 CD4^{+} T cells shown immediately above in A. Plotted are the mean values (±SEM) of 2 technical replicates, from 2 experiments with 5 mice per group per experiment.

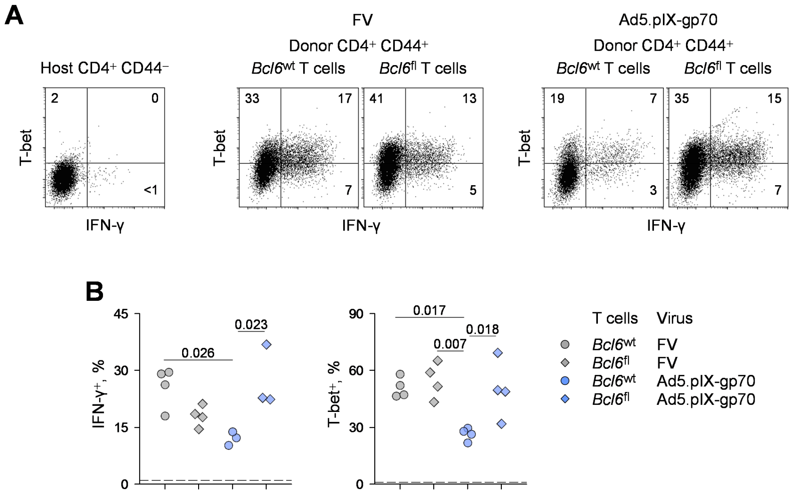

Figure S9. Related to Figure 3; Effect of *Bcl6* deletion on CD4^{+} effector development

(A) Flow cytometric detection of intracellular IFN-γ and T-bet expression in host naïve (CD44^{-}) or env-reactive (CD44^{+}) *Bcl6*^{wt} or *Bcl6*^{fl} donor EF4.1 CD4^{+} T cells, in the spleens of recipient mice, 7 days after adoptive transfer and FV infection or Ad5.pIX-gp70 immunization.

(B) Frequency of IFN-γ^{+} and T-bet^{+} cells in env-reactive *Bcl6*^{wt} or *Bcl6*^{fl} donor EF4.1 CD4^{+} T cells from the same recipients. Each symbol is an individual mouse from one representative of two experiments.

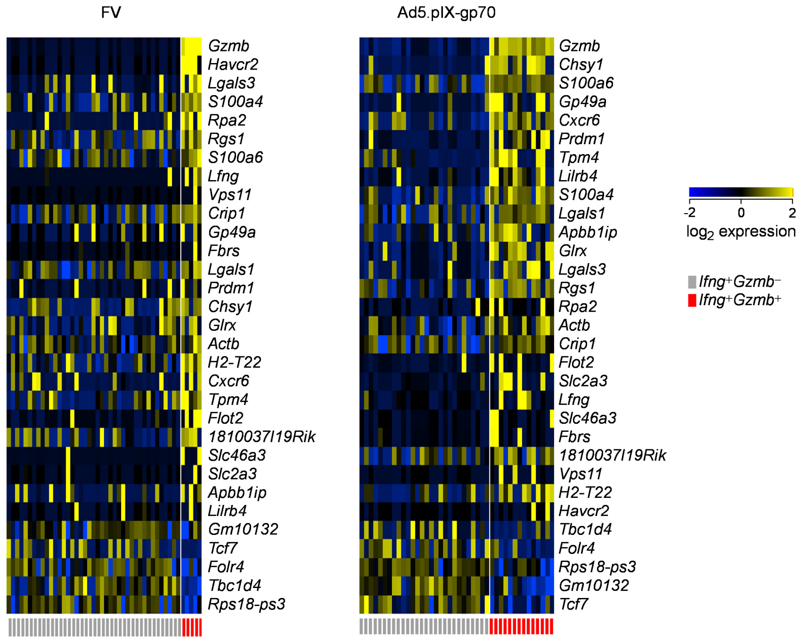

Figure S10. Related to Figure 5; CD4$^+$ CTL and Th1 cells are transcriptionally distinct: effect of priming virus

Heat-maps of gene expression, assessed by single-cell RNA sequencing, comparing *Ifng*$^+$*Gzmb*$^+$ and *Ifng*$^+$*Gzmb*$^-$ subsets of env-reactive donor EF4.1 CD4$^+$ T cells purified from the spleens of recipient mice, 7 days after adoptive transfer either FV infection (p=0.064) (*left*) or Ad5.pIX-gp70 immunization (p=0.00154) (*right*). Both *Bcl6*^{wt} and *Bcl6*^{fl} CD4$^+$ T cells are included.

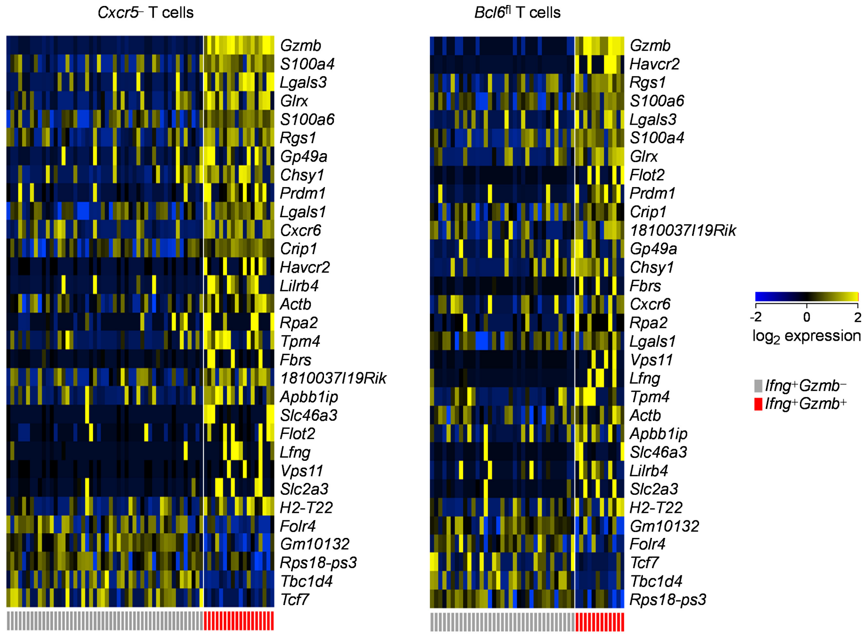

Figure S11. Related to Figure 5; CD4$^+$ CTL and Th1 cells are transcriptionally distinct: exclusion of Tfh cells

Heat-maps of gene expression, assessed by single-cell RNA sequencing, comparing *Ifng*$^+$*Gzmb*$^+$ and *Ifng*$^+$*Gzmb*$^-$ subsets of env-reactive donor EF4.1 CD4$^+$ T cells purified from the spleens of recipient mice, 7 days after adoptive transfer and priming. Cells from both FV infection and Ad5.pIX-gp70 immunization are included. Tfh cells were omitted from this analysis either by excluding cells that express *Cxcr5* (p=0.0038) (*left*) or by including only cells that have conditionally lost *Bcl6* (p=0.0345) (*right*), and therefore the ability to differentiate into Tfh cells.

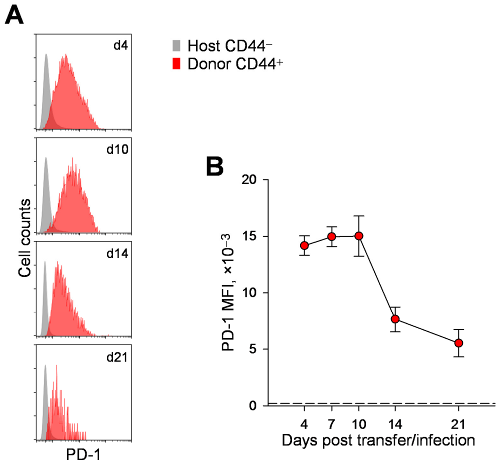

Figure S12. Related to Figure 6; Time course of PD-1 expression in CD4$^+$ T cells responding to FV infection

(A) Flow cytometric detection of surface PD-1 expression in host naïve (CD44$^-$) or env-reactive (CD44$^+$) donor EF4.1 CD4$^+$ T cells, in the spleens of recipient mice, at the indicated days after adoptive transfer and FV infection.

(B) MFI of PD-1 staining in the same cells as in A. Plotted are the mean values (±SEM) from 1 experiment with 4 mice per time-point.

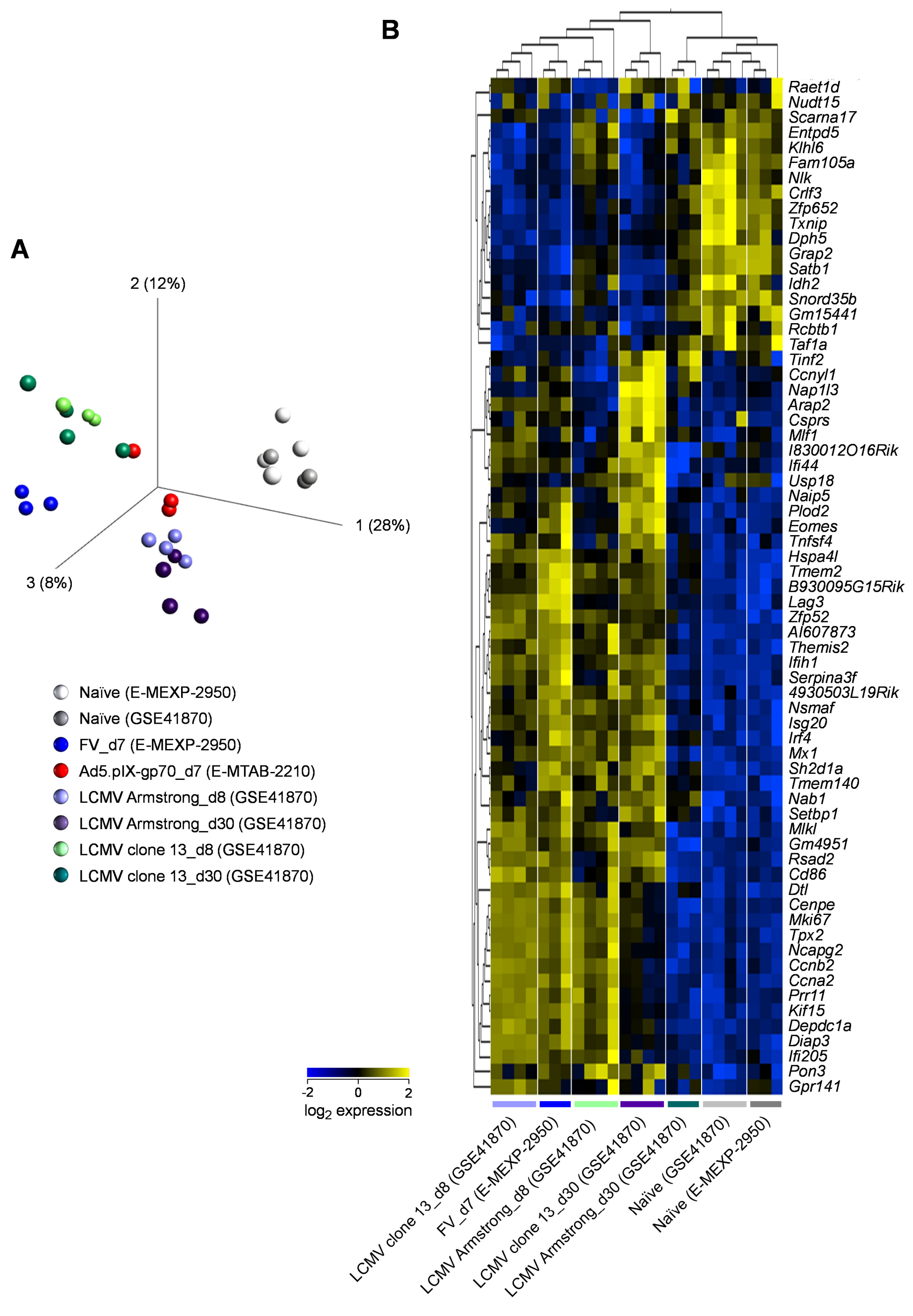

Figure S13. Related to Figure 6; Comparison of FV-primed effector and exhausted CD4$^+$ T cells

(A) Principal component analysis (PCA) of the transcriptional profiles of naïve and FV-primed effector (d7) EF4.1 CD4$^+$ T cells (study E-MEXP-2950), Ad5.pIX-gp70-primed effector (d7) EF4.1 CD4$^+$ T cells (study E-MTAB-2210) and naïve, effector (d8) and exhausted (d30) CD4$^+$ T cells primed by either LCMV Armstrong or clone 13 (study GSE41870). LCMV clone 13-primed CD4$^+$ T cells on day 30 of infection typify the exhaustion profile. All transcriptional profiles were obtained with the Affymetrix Mouse Gene 1.0 ST Array and can be accessed with the indicated accession numbers.

(B) Hierarchical clustering of the same samples according to the expression of the list of genes reported to signify the exhaustion phenotype (Crawford 2014; Table S3). FV-primed effectors cluster together with other effector subsets.

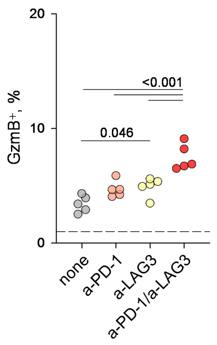

Figure S14. Related to Figure 6; PD-1 and LAG3 cooperatively inhibit CD4$^+$ CTL development

Frequency of intracellular GzmB$^+$ cells in bulk env-reactive donor EF4.1 CD4$^+$ T cells in the spleens of recipient mice, 7 days after adoptive transfer and FV infection. The indicated groups additionally received treatment with either PD-1 or LAG3 blocking antibodies separately or combined. Each symbol is an individual recipient.

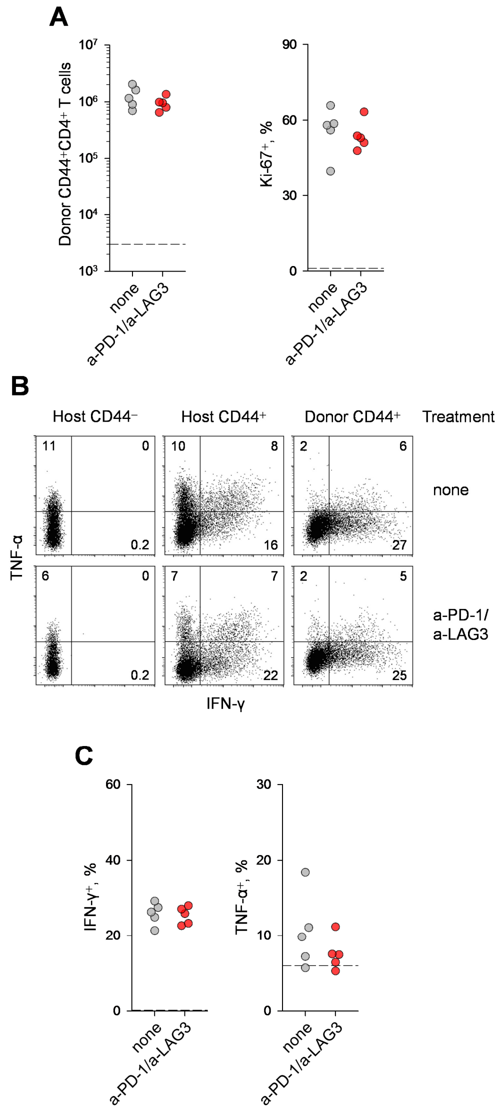

Figure S15. Related to Figure 6; Effect of PD-1 and LAG3 blockade on CD4+ effector development

(A) Absolute numbers of env-reactive donor EF4.1 CD4+ T cells (*left*) and frequency of Ki67+ cells within these cells (*right*) recovered from the spleens of recipient mice, 7 days after adoptive transfer and FV infection, in the presence or absence of PD-1 or LAG3 blocking antibodies.

(B) Flow cytometric detection of intracellular IFN-γ and TNF-α expression in host naïve (CD44-) or env-reactive (CD44+) donor EF4.1 CD4+ T cells, in the spleens of recipient mice, 7 days after adoptive transfer and FV infection, in the presence or absence of PD-1 or LAG3 blocking antibodies.

(C) Frequency of IFN-γ+ and TNF-α+ cells in the same cells as in B. In A and C, each symbol is an individual mouse and dashed lines represents host naïve CD4+ T cells.

Supplementary Table 1: Differences in gene expression between Gzmb+ and Gzmb- cells.

Please see Excel file

Supplementary Table 2: PCR primers used in this study

Gene	Forward primer	Reverse primer
Zbtb7b	ATGGGATTCCAATCAGGTCA	TTCTTCCTACACCCTGTGCC
Runx3	ACAGCATCTTTGACTCCTTCC	TGTTCTCGCCCATCTTGC
Tcf7	CAATCTGCTCATGCCCTACC	CTTGCTTCTGGCTGATGTCC
Gzmb	CCTCCTGCTACTGCTGACCT	TAAGGCCATGTAGGGTCGAG
Prdm1	ACATAGTGAACGACCACCCCTG	CTTACCACGCCAATAACCTCTTTG
Bcl6	CCTGTGAAATCTGTGGCACTCG	CGCAGTTGGCTTTTGTGAC
Gata3	ACAGAAGGCAGGGAGTGTGTGAAC	TTTTATGGTAGAGTCCGCAGGC
Tbx21	CAATGTGACCCAGATGATCG	GCGTTCTGGTAGGCAGTCAC
Ifng	GATGCATTCATGAGTATTGCCAAGT	GTGGACCACTCGGATGAGCTC
Hprt	TTGTATACCTAATCATTATGCCGAG	CATCTCGAGCAAGTCTTTCA

SUPPLEMENTAL EXPERIMENTAL PROCEDURES

Retroviral infection and immunization

The FV used in this study was a retroviral complex of a replication-competent B-tropic F-MLV and a replication-defective spleen-focus forming virus (SFFV). Stocks were propagated *in vivo* and prepared as 10% w/v homogenate from the spleen of 12-day infected BALB/c mice, as previously described (Marques et al., 2008). Mice received an inoculum of ~1,000 spleen focus-forming units of FV by intravenous injection. Stocks of F-MLV-N helper virus were grown in *Mus dunni* fibroblast cells. Mice received an inoculum of ~10^4 infectious units of F-MLV-N by intravenous injection. Ad5.pIX-gp70 stocks were prepared at a titer of 9×10^9 viral genomes ml^{-1} by infection of 293A cells (Thorborn et al., 2014). Approximately 5×10^8 Ad5.pIX-gp70 viral genomes per mouse were administered intravenously. The mCMV vector expressing F-MLV env (mCMV.env) was constructed by inserting the F-MLV *env* open reading frame, under the control of the human CMV major IE promoter/enhancer, into the m157 open reading frame of mCMV in the MCK-2 repaired background cloned into a bacterial artificial chromosome (BAC) (Jordan et al., 2011). The recombinant mCMV.env was reconstituted from BAC DNA by transfection into permissive fibroblasts. Persistence of the mCMV vector, as well as retention of the F-MLV *env* transgene, were confirmed by PCR in mCMV.env genomes isolated from the salivary glands of infected mice at 21 days post infection. Mice received 2×10^5 plaque-forming units of mCMV.env intraperitoneally. All stocks were free of Sendai virus, Murine hepatitis virus, Parvoviruses 1 and 2, Reovirus 3, Theiler's murine encephalomyelitis virus, Murine rotavirus, Ectromelia virus, Murine cytomegalovirus, K virus, Polyomavirus, Hantaan virus, Murine norovirus, Lymphocytic

choriomeningitis virus, Murine adenoviruses FL and K87, *Mycoplasma* sp. and Lactate dehydrogenase elevating virus. FBL-3 tumor challenge was carried out by intravenous injection of 3×10^6 FBL-3 cells (Klarnet et al., 1989). For peptide immunization, mice received an intraperitoneal injection of a total of 12.5 nmol of synthetic $env_{122-141}$ peptide mixed in Sigma Adjuvant System. Where indicated, recipient mice also received blocking antibodies against PD-1 (10 mg/kg, clone RMP1) and LAG3 (20mg/kg, clone C9B7W) (both from BioXCell, West Lebanon, NH, USA) injected intraperitoneally on days 0, 2 and 4 of FV infection.

Single-cell RNA sequencing

Cells were then applied to the Fluidigm C1 chip (Fluidigm, San Francisco, CA, USA) and captures were imaged for manual grading. Captures were split into 5 categories: no capture (0), debris (D), single cell (1), multiple cells (M), and non-standard single cell (NS - including large, small, potentially damaged or dying). Libraries were constructed using the SMART-Seq v4 Ultra Low RNA Kit for the Fluidigm C1 System (Clontech, Mountain View, CA, USA), following the manufacturer's instructions, and validated with the use of spiked-in RNA species (Ambion ArrayControl RNA Spikes, Thermo Fisher Scientific, Waltham, MA USA). Reads (2×50 bp paired-end), generated with the Illumina HiSeq 2000 (Illumina, San Diego, CA, USA), were adapter- and quality-trimmed using Trimmomatic 0.32 (Bolger et al., 2014). Resulting reads pairs with a read <30 nucleotides in length were discarded, resulting in a median pair retention of 94%. Pairs were mapped to the mouse genome (GRCm38.78) with the splice-aware aligner HISAT (Kim et al., 2015) and gene-level counts were produced with featureCounts (Liao et al., 2014). To streamline downstream analysis, any genes not found

expressed in at least 1% of samples were discarded, resulting in 11463 genes in the subsequent analysis. Whilst only morphologically normal single cells are included in the '1' capture category, any library preparations may suffer as a result of poor lysis of the cell, or if, whilst morphologically normal, a cell was dead or dying. To prevent such libraries from impacting the analysis and to allow inclusion of transcriptionally similar cells from the 'NS' capture category, a classifier was build using all cells. DESeq2 (Love et al., 2014) was used for initial library size normalization and individual differential expression (DE) analysis of '0' and 'D' captures against combined '1', 'NS', and 'M' captures. The intersect of the top 250 DE genes for these capture categories was taken, giving a list of 76 genes that were used to build a supervised SVM classifier using the R e1071 package (wrapping LIBSVM) (Chang and Lin, 2011) that was auto-tuned to the data. Excluding '0', 'D', and 'M' capture categories, the SVM was applied to '1' and 'NS' cells, providing a numerical likelihood of a sample having better correspondence to a '0' or 'D' capture. Any cells with correspondence 50% or larger were excluded from further analysis. Plots of percentage alignment against number of genes identified for cells passing and failing these filters confirmed that excluded cells frequently had poor alignment rates and lower numbers of genes identified as expressed, although overall read count did not correspond to either of these metrics. Subsequently, samples with <15% alignment rate and those with <1000 genes identified as expressed were additionally excluded. Retained cells from the '1' and 'NS' capture categories were again passed to DESeq2 and DE analysis was performed between various conditions. Normalised count data was exported at this point for further analysis and plotting using Qlucore Omics Explorer (Qlucore, Lund, Sweden). The European Nucleotide Archive (ENA) accession number for the single-cell RNA sequences reported in this paper is PRJEB14043.

Cytotoxicity assays

In vivo cytotoxicity was assessed by the specific killing of peptide-pulsed targets of $CD4^+$ CTLs. Single-cell suspensions were prepared from the spleens and lymph nodes of donor $CD45.1^+$ or $CD45.2^+$ WT mice and B cells ($B220^+$ cells) were purified using immunomagnetic positive selection (StemCell Technologies). B cells from only one type of donor were incubated *in vitro* for 2 hours with 1µM $env_{122-141}$ peptide. After washing, pulsed and non-pulsed B cells from the two types of donor were mixed at equal ratios and co-injected into the indicated recipients (5×10^6 of each per recipient). The ratio between pulsed and non-pulsed B cells was measured by flow cytometry in the spleens of recipients 24 hours after B cell transfer. The percentage difference in this ratio was expressed as percentage killing. *In vitro* cytotoxicity was measured using the GranToxiLux-PLUS kit (Oncoimmunin, Gaithersburg, MD, USA), according to the manufacturer's instructions. Briefly, WT splenocytes were used as target cells and were either incubated *in vitro* for 2 hours with 1µM $env_{122-141}$ peptide (pulsed) or not (non-pulsed). Effector EF4.1 $CD4^+$ T cells were purified by cell sorting from the spleens of FV infected or Ad5.pIX-gp70 vaccinated recipients on day 7 of the response. Target cells were identified by labelling with a target fluorescent probe (TFL-4) and with a nuclear fluorescent labelling probe (NFL1), to exclude cells that had died before the start of the assay. Effector and target cells were mixed at a ratio of 5:1 and co-incubated in the presence of a FITC-conjugated GzmB substrate for 2 hours. Cytotoxic activity was detected by the cleavage of the substrate, which released FITC and thus rendered target cells fluorescent.

SUPPLEMENTAL REFERENCES

Bolger, A.M., Lohse, M., and Usadel, B. (2014). Trimmomatic: a flexible trimmer for Illumina sequence data. Bioinformatics *30*, 2114-2120.

Chang, C.-C., and Lin, C.-J. (2011). LIBSVM: A library for support vector machines. ACM Trans. Intell. Syst. Technol. *2*, 1-27.

Jordan, S., Krause, J., Prager, A., Mitrovic, M., Jonjic, S., Koszinowski, U.H., and Adler, B. (2011). Virus progeny of murine cytomegalovirus bacterial artificial chromosome pSM3fr show reduced growth in salivary Glands due to a fixed mutation of MCK-2. J. Virol. *85*, 10346-10353.

Kim, D., Langmead, B., and Salzberg, S.L. (2015). HISAT: a fast spliced aligner with low memory requirements. Nat. Methods *12*, 357-360.

Klarnet, J.P., Kern, D.E., Okuno, K., Holt, C., Lilly, F., and Greenberg, P.D. (1989). FBL-reactive CD8+ cytotoxic and CD4+ helper T lymphocytes recognize distinct Friend murine leukemia virus-encoded antigens. J. Exp. Med. *169*, 457-467.

Liao, Y., Smyth, G.K., and Shi, W. (2014). featureCounts: an efficient general purpose program for assigning sequence reads to genomic features. Bioinformatics *30*, 923-930.

Love, M.I., Huber, W., and Anders, S. (2014). Moderated estimation of fold change and dispersion for RNA-seq data with DESeq2. Genome Biol. *15*, 550.

Marques, R., Antunes, I., Eksmond, U., Stoye, J., Hasenkrug, K., and Kassiotis, G. (2008). B lymphocyte activation by coinfection prevents immune control of friend virus infection. J. Immunol. *181*, 3432-3440.

Thorborn, G., Ploquin, M.J., Eksmond, U., Pike, R., Bayer, W., Dittmer, U., Hasenkrug, K.J., Pepper, M., and Kassiotis, G. (2014). Clonotypic composition of the CD4+ T cell response to a vectored retroviral antigen is determined by its speed. J. Immunol. *193*, 1567-1577.
